# Hyperphosphorylation as a Defense Mechanism to Reduce TDP-43 Aggregation

**DOI:** 10.1371/journal.pone.0023075

**Published:** 2011-08-05

**Authors:** Huei-Ying Li, Po-An Yeh, Hsiu-Chiang Chiu, Chiou-Yang Tang, Benjamin Pang-hsien Tu

**Affiliations:** 1 Molecular Medicine Program, Taiwan International Graduate Program, Institute of Biomedical Sciences, Academia Sinica, Taipei, Taiwan; 2 Institute of Biochemistry and Molecular Biology, School of Life Sciences, National Yang-Ming University, Taipei, Taiwan; 3 Institute of Biomedical Sciences, Academia Sinica, Taipei, Taiwan; 4 Institute of Molecular Biology, Academia Sinica, Taipei, Taiwan; Nathan Kline Institute and New York University School of Medicine, United States of America

## Abstract

Several neurodegenerative diseases including amyotrophic lateral sclerosis (ALS) and frontotemporal lobar degeneration with ubiquitinated inclusions (FTLD-U) are characterized by inclusion bodies formed by TDP-43 (TDP). We established cell and transgenic Drosophila models expressing TDP carboxyl terminal fragment (ND251 and ND207), which developed aggregates recapitulating important features of TDP inclusions in ALS/FTLD-U, including hyperphosphorylation at previously reported serine^403,404,409,410^ residues, polyubiquitination and colocalization with optineurin. These models were used to address the pathogenic role of hyperphosphorylation in ALS/FTLD-U. We demonstrated that hyperphosphorylation and ubiquitination occurred temporally later than aggregation in cells. Expression of CK2α which phosphorylated TDP decreased the aggregation propensity of ND251 or ND207; this effect could be blocked by CK2 inhibitor DMAT. Mutation of serines^379,403,404,409,410^ to alanines (S5A) to eliminate phosphorylation increased the aggregation propensity and number of aggregates of TDP, but mutation to aspartic acids (S5D) or glutamic acids (S5E) to simulate hyperphosphorylation had the opposite effect. Functionally, ND251 or ND207 aggregates decreased the number of neurites of Neuro2a cells induced by retinoic acid or number of cells by MTT assay. S5A mutation aggravated, but S5E mutation alleviated these cytotoxic effects of aggregates. Finally, ND251 or ND251S5A developed aggregates in neurons, and salivary gland of transgenic Drosophila, but ND251S5E did not. Taken together, our data indicate that hyperphosphorylation may represent a compensatory defense mechanism to stop or prevent pathogenic TDP from aggregation. Therefore, enhancement of phosphorylation may serve as an effective therapeutic strategy against ALS/FTLD-U.

## Introduction

Amyotrophic lateral sclerosis (ALS) and frontotemporal lobar degeneration with ubiquitinated inclusions (FTLD-U) have been recognized as two entities within a spectrum of neurodegenerative diseases in light of overlapped clinical presentations and shared neuropathological lesions characterized by ubiquitinated inclusion bodies first recognized in a subset of patients [1], [2]. Recently, Tar DNA-binding protein (*TARDBP*) gene-encoded product TDP-43 (TDP) was discovered as the major component of the signature inclusion bodies in ALS/FTLD-U [3], [4], and several other neurodegenerative diseases, collectively called TDP proteinopathy [5], [6]. TDP pathology also co-existed in Alzheimer's disease [7], dementia with Lewy bodies [8], and Huntington's disease [9]. These studies have firmly established TDP as one of the important protein molecules in the pathogenesis of several neurodegenerative diseases.

TDP is a nuclear RNA-binding protein. It affects HIV infectivity [10], promotes exon-9 skipping of the *CFTR* transcript, and is important for neurological function [11], [12], [13], [14], which may be linked to its versatile roles involved in exon-7 inclusion of the *SMN* transcript [15], neurofilament light mRNA stabilization [16], regulation of mRNAs dynamics in synapses [17], and regulation of expression of let-7b microRNA which in turn modulates several important transcripts involved in neurodegeneration and synapse formation [18].

TDP inclusions were found in neurological diseases caused by mutations in genes valosin-containing protein [19], [20], progranulin [21], [22], [23], [24], dynactin [25] and optineurin [26]. Mutations in *TARDBP* gene were identified in familial ALS [27], [28], [29], [30], [31], confirming its causal role in the pathogenesis of ALS. TDP in ALS/FTLD-U undergoes pathognomonic alterations, including cytoplasmic translocation, putative carboxyl terminal cleaved fragment [32], and hyperphosphorylation [3], [4]. Recently, elucidation of the role these changes played in TDP aggregation took the central stage. Cytoplasmic translocation and cleavage were shown to promote TDP inclusions [32], [33], [34], [35]; however, hyperphosphorylation remains less characterized.

Dr. Hasegawa and colleagues elegantly showed that ser379, ser403/ser404 and ser409/ser410 residues of tdp were phosphorylated in als/ftld-u inclusions, presumably by casein kinases (cks) 1 and 2 [36], [37], [38], which has been validated in subsequent studies [39] and other diseases [9], [40]. He proposed hyperphosphorylation as a precursor change toward tdp inclusions. In this study, our data suggested alternatively that hyperphosphorylation was a compensatory mechanism against tdp aggregation.

## Materials and Methods

### Generation of TDP constructs

p*eGFPTDP* was generated by cloning BamHI/HindIII fragment of full length TDP (fTDP) into *peGFPC3* (Clontech) after RT-PCR from HEK293T cells (Cat #CRL-11268 from American Type Culture Collection, VA, USA) using SuperScript III Reverse Transcription kit (Invitrogen) and primers: 5′ATCGATGGATCCTCTGAATATATTCGGGTAAC3′; 5′ATCGATAAGCTTCTACATTCCCCAGCCAGAAG3′. p*eGFPND104* and p*eGFPND251* were generated using p*eGFPTDP* as template with primers 5′GGAAGCTTGCCACCATGTCTGAATATATTCGG3′ and 5′GAAGCTTGCCACCATGGATTTAATAGTGTTG3′; 5′GGAAGCTTGCCACCATGGGAATCAGCGTTCAT3′ and 5′CGATGTCGACCTACATTCCCCAGCCAGAAG3 ′. p*eGFPND207* was generated using *peGFPTDP* as template and primers 5′CGGGAGTTCTTCTCTCAGTAC3′ and 5′AAGCTTGAGCTCGAGATCTG3′. S→A and S→E mutants constructs were generated by sequential PCRs using the p*eGFPTDP*, p*eGFPND207* or p*eGFPND251* as templates with primers: 5′AATGCTGGTGCAGCAATTGGTTG3′ or, 5′AATGAAGGTGCAGCAATTGGTTG3′ with 5′AGAGCCACTATAAGAGTTATTTC3′; 5′AATGGAGGCTTTGGCGCAGCCATGGATTCTAAG3′ or 5′CTTAGAATCCATGGCTGCGCCAAAGCCTCCATT3′ with 5′AATGGAGGCTTTGGCGAAGAGATGGATTCTAAG3′. To generate emGFPND207 and its mutants, TDPwt first cloned into *pCol1a1-emGFP/Blue Cla pA* vector (a gift from Dr. Alexander C. Lichter) to generate *pCol1a1-emGFPTDPwt*. *pCol1a1-emGFPND207 was further generated* with primers: 5′CGGGAGTTCTTCTCTCAGTAC3′ and 5′AAGCTTGAGCTCGAGATCTG3′. *pREV-TRE-emGFPND207* was generated by subcloning emGFPND207 fragment released from *pCol1a1-emGFPND207* with SalI/Hind III into the same sites of *pREV-TRE* vector (Clontech).

### Antibodies

The rabbit polyclonal GFP antiserum was purchased from Clontech (CA, USA); the rabbit polyclonal antisera pS403/404 and pS409/410, from Cosmo Bio Co. (Tokyo, Japan); the anti-GAPDH and anti-ubiquitin mouse monoclonal antibodies (mAbs), from Chemicon (MA, USA); the anti-Flag (M2) mAb, from Sigma (MO, USA); the goat anti-HA tag polyclonal antibody, from GenScript (NJ, USA); the anti-Myc (Myc.A7) mAb, from Anogen (ON, Canada); the anti-optineurin rabbit polyclonal antiserum, from Cayman Chemical (MI, USA). The self-generated rabbit TDP-43 polyclonal antiserum was raised against amino acids 352∼367 of human TDP (LTK Biolaboratories, Taiwan). All the peroxidase-conjugated secondary antibodies were purchased from Jackson ImmunoResearch (PA, USA).

### Cell preparation

HEK293T cells and Neuro2a cells (Cat #CRL-11268 and CCL-131 from American Type Culture Collection, VA, USA) were maintained in DMEM (Gibco) with 10% fetal bovine serum and 1% penicillin-streptomycin (Gibco). For fluorescent and confocal microscopy studies, 1.5×10^5^ HEK293T cells or 5×10^4^ Neuro2a cells were plated on a coverslip and transfected using Lipofectamine 2000 (Invitrogen) for specified times or 48 hrs. Cells were then fixed with 4% paraformaldehyde/phosphate-buffered saline (PBS) for 15 minutes at room temperature, then penetrated with 0.2% Triton X-100/PBS for 10 minutes, stained with appropriate antibodies and counterstained with DraQ5 (Cat #4084S, Millipore, MA, USA) or DAPI for 10 minutes.

### Analyses of aggregates

For quantitative analyses of aggregates, all samples were visualized under the Nikon Eclipse TE-2000U microscope, and the images were captured and processed by a SPOT RT3 digital camera and software (Diagnostic Instruments, MI). Five representative fields per sample were taken and analyzed by MetaMorph software (Molecular Devices, Downingtown, PA). GFP signal was gated to exclude non-transfected cells, and the images were then superimposed with corresponding DAPI images. The Metamorph was used to count the total number of transfected cells. For aggregate analyses, the GFP images were visually adjusted to determine a common threshold across all samples to eliminate diffuse or non-aggregated signals. The area and number of individual aggregate were calculated with the Integrated Morphometry Analysis of Metamorph. On average, ∼2000 cells per sample were counted. The average size of inclusions was calculated by the formula: total areas of inclusions/total number of inclusions. Statistical significance was analyzed with the student *t*-test.

### Isolation of ND251 aggregates

ND251 aggregates vs. eGFP control were isolated as described by Mitsui et al. [41] with some modifications. Briefly, transfected HEK293T cells or Neuro2a cells were lysed with RIPA buffer (50 mM Tris-HCl pH 7.5, 150 mM NaCl, 1% NP-40, 0.1% SDS) plus protease inhibitors, phosphatase inhibitors and 1 mg/mL DNase I (all from Sigma) on ice for 30 minutes with shaking. Cell lysates were harvested, sonicated for 20 seconds, and then loaded into FACSAria cell sorter (BD Biosciences). The flow rate was adjusted to ∼4000 events/second, and the intensity of GFP fluorescence was used to gate the cell lysates. The collected materials from P1 and P2 fractions were centrifuged at 14 K rpm for 10 min at 4°C, and then observed by Eclipse TE-2000U inverted fluorescence microscope. The materials were then solubilized in 100 µL urea buffer (100 mM Tris-Cl pH 6.8, 4% SDS, 20% glycerol, 0.05% Bromophenol blue and 8 M Urea) for further studies.

### Assay of the solubility of Hyper- and un-phosphorylated ND251 from isolated inclusions

ND251 aggregates were isolated as described in the previous section. About 3.2×10^7^ particles were spun down by 1,3000 rpm (SW41 rotor, Beckman) for 10 min. Pellets were solubilized and sonicated in 600 µL of buffer (8 M Urea/50 mM Tris-Cl pH 7.5/150 mM NaCl/1.43% β-ME). 100 µL aliquots were inserted into the Thermo Slide-ALyzer Mini Dialysis Unit (Cat No. 69550) and dialyzed against 500 mL buffer (50 mM Tris-Cl pH 7.5/150 mM NaCl) containing 0, 0.5, 1, 2 or 4 M Urea buffer for 16 hrs. The dialyzed samples were centrifuged at 100,000 g (TLA100.2 rotor, Backman) for 2 hrs. Supernatants (soluble fractions) were saved and the pellets (insoluble fractions) were dissolved in 200 µL 2× SDS/8 M Urea buffer. 1/80 of RIPA and urea samples were electrophoresed by 10% SDS-PAGE. Immunoblotting were performed with self-generated anti-TDP antiserum which recognized both Hyper- and un-phosphorylated ND251. Digital images were captured with LAS-3000 imaging system (Fujifilm, Japan) and the intensity of individual bands was quantified by ImageQuant (Molecular Dynamics). The solubility was obtained by the formula: intensity of protein in supernatant/the intensity of protein in supernatant+that in pellet.

### Protein solubility and immunoblotting

Transfected HEK293T or Neuro2a cells were sequentially extracted with ice-cold RIPA buffer supplemented with protease inhibitors, and phosphatase inhibitors. Cell lysates were sonicated for 20 seconds and then centrifuged at 13 K rpm for 10 minutes at 4°C. The supernatants were designated soluble (R) fraction. The RIPA extraction was repeated once again. The insoluble pellets were then sonicated and solubilized in urea buffer. After centrifuge, the supernatants were insoluble (U) fraction. 1/20 of RIPA and urea samples were resolved by 10% SDS-PAGE, transferred to polyvinylidene difluoride membrane (Millipore), and then probed with primary and secondary antibodies to be visualized with Immobilon chemiluminescent reagents (Millipore). Digital images were captured with LAS-3000 imaging system (Fujifilm, Japan) and the intensity of individual bands was quantified by ImageQuant (Molecular Dynamics).

### Neurite outgrowth assay

Transfected Neuro2a cells were grown in culture medium containing 10 µM retinoic acid (Sigma) and 1% fetal bovine serum to allow for differentiation for 48 hrs. Differentiated cells were observed under Eclipse TE-2000U microscope, and corresponding phase contrast and fluorescent images were captured by a SPOT RT3 digital camera. Only cells with green fluorescence signal were included for further analysis. The neurites were defined as cellular processes ≧2 times of diameter of cell body. On average, ∼200 cells were counted per sample and experiment sets were done in triplicates. The statistical significance was analyzed by the Student's *t*-test.

### Cell proliferation assay (MTT assay)

In Neuro2a cells, *pTet-off* (Clontech) was co-transfected with either *pemGFP*, *pemGFPND207*, *pemGFPND207S5A* or *pemGFPND207S5E* to achieve highest expression of truncated TDP-43 aggregates. After transfection for 6 hours, 2000 cells were seeded into 96 wells with 100 µL culture medium. At different time point, 10 µL of 5 mg/ml MTT solution (3-(4, 5-dimethylthiazol-2-yl)-2, 5-diphenyl tetrazolium bromide, Sigma M2128) were added to each well. After incubation at 37°C for 3 hours, the insoluble formazan product was dissolved in 0.1 N HCl in anhydrous isopropanol and absorbance at λ = 570 nm was read. The OD value of truncated TDP-43 groups was divided by the OD value of emGFP control group and results presented as percentage of control group (as 100%).

### Generation of Transgenic Drosophila models

*ywhsFLP*; *Actin-Gal4>y+>UAS-β-galactosidase* [42], *UAS-DsRed*, *GMR-Gal4* and *elav-Gal4/Cyo* were obtained from Bloomington Stock Center. *UAS-ND251*, *UAS-ND251S5A* and *UAS-ND251S5E* (all tagged with eGFP) transgenic flies were generated by injecting pUAST constructs to fly eggs carrying transposase (Δ2–3).

### Random expression of ND251 variants in salivary gland

*ywhsFLP*; *Actin-Gal4>y+>UAS-β-galactosidase* flies were crossed with transgenic flies with *UAS-ND251* or its variants and cultured at 25°C. When the progeny grew to second larval stage, vials were heat-shocked at 37°C for 30 minutes to induce random expression. Then, larvae of the third instar stage were examined by Zeiss fluorescence dissecting microscope, and the salivary glands were dissected out for further analysis with LSM 510 Meta confocal microscope (Zeiss).

### Pan-neuronal expression of ND251 variants in transgenic flies

Male flies of *UAS-DsRed*; *elav-Gal4/+* were crossed with *UAS-ND251* variants transgenic flies. Third instar larvae were examined by dissecting fluorescence microscope, and dissected to eliminate guts and other organs. The remnants were fixed with paraformaldehyde and counterstained with DAPI for further analysis with confocal microscope.

## Results

### Truncated TDP having higher aggregation propensity than full length TDP

**Figure 1:**
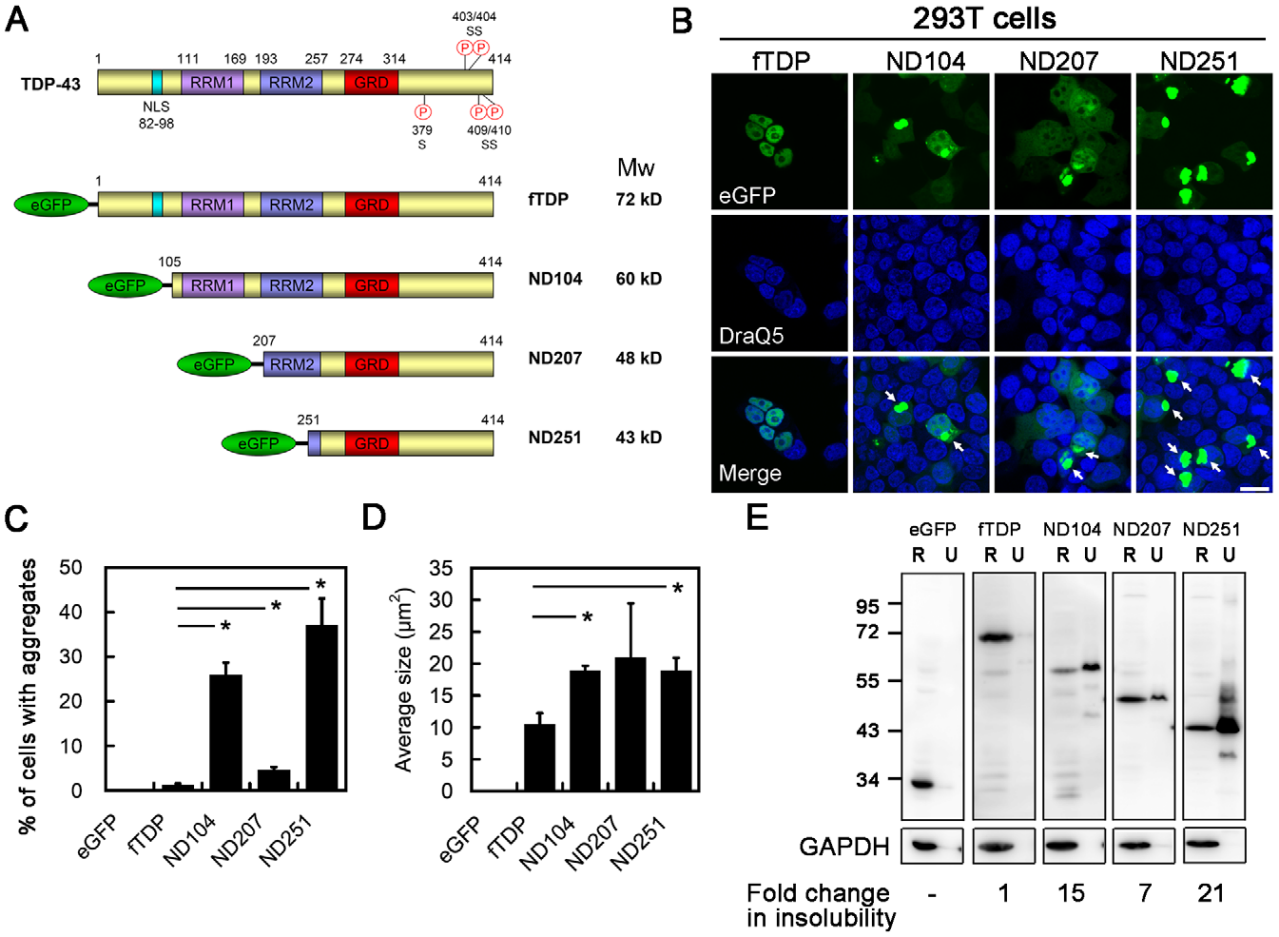
Formation of inclusions by truncated TDP. (*A*) A diagram illustrating the domain structure of TDP along with various eGFP-fused N-terminal truncated constructs used in this study. (*B*) Micrograph montage of 293T cells expressing fTDP and truncated forms of TDP (ND104, ND207 and ND251). Inclusion bodies formed by truncated TDP-43 were indicated by arrows. Scale bar = 20 µm (*C*) Quantitative analysis of the number of inclusions per 1000 transfected cells. Please note that ND251 formed the highest number of inclusions among all (P<0.00001). (*D*) Average size (µm^2^) of TDP inclusions measured by Metamorph software. The inclusions formed by truncated TDP were larger than those of fTDP in size. *, P<0.05. (*E*) Western blot analysis of soluble and insoluble portions of various forms of TDP protein. An increase in TDP partitioned into urea (U) fraction was consistently observed in all three truncated TDP samples. GAPDH was used as loading control. The number indicated the fold change in insoluble fraction of TDP quantified by ImageQuant. R: RIPA buffer.

eGFP-fused full length (fTDP) and three truncated forms of TDP with N-terminal 104 (ND104), 207 (ND207) and 251 (ND251) amino acids deleted were used in this study to investigate the differential effect of individual domains of TDP on aggregation propensity (Fig. 1A). ND207 was specifically created to model after the 25 kDa carboxyl terminal cleaved fragments in ALS/FTLD-U cases [32]. fTDP was mainly diffusely distributed in nucleoplasm like the endogenous TDP, but aggregates were found in a low percentage (1.3±0.3%) of transfected HEK293T cells. In contrast, ND104, ND207 and ND251 were expressed more in cytoplasm than in nuclei for lack of nuclear localization signal (NLS), and formed aggregates in 25.9±2.8%, 4.6±0.7% and 37.0±6.0% of transfected cells, respectively (Fig. 1B and 1C). As expected, ND104, ND207 and ND251 aggregates localized mainly in the cytoplasm (80.8%, 81.1% and 95.9% respectively) due to the deletion of the nuclear localization signal (NLS) located in the N-terminus. The aggregates formed by the individual truncated TDP did not differ in size (18.8±2.1 µm^2^ in average), and were significantly bigger than those formed by fTDP (10.4±1.8 µm^2^) (Fig. 1D). Western blot analyses revealed changes in the solubility of these transgenic proteins corresponding to their aggregation propensities. As shown in Fig. 1E, fTDP was highly soluble in the RIPA buffer with only trace amount of insoluble (aggregated) protein found in urea fraction. In contrast, all truncated forms of TDP exhibited substantial increases in their insoluble pools. Notably, the pattern of insoluble ND251 revealed a shorter fragment and high molecular weight smear, reminiscent of that of TDP inclusions in ALS/FTLD-U [3], [4]. These results showed that both fTDP and truncated TDP species formed aggregates, but the latter had higher aggregation propensity than the former, and the carboxyl terminus to be essential for TDP aggregation in the cell models.

### ND251 aggregates recapitulating important features of TDP inclusions in ALS/FTLD-U

**Figure 2:**
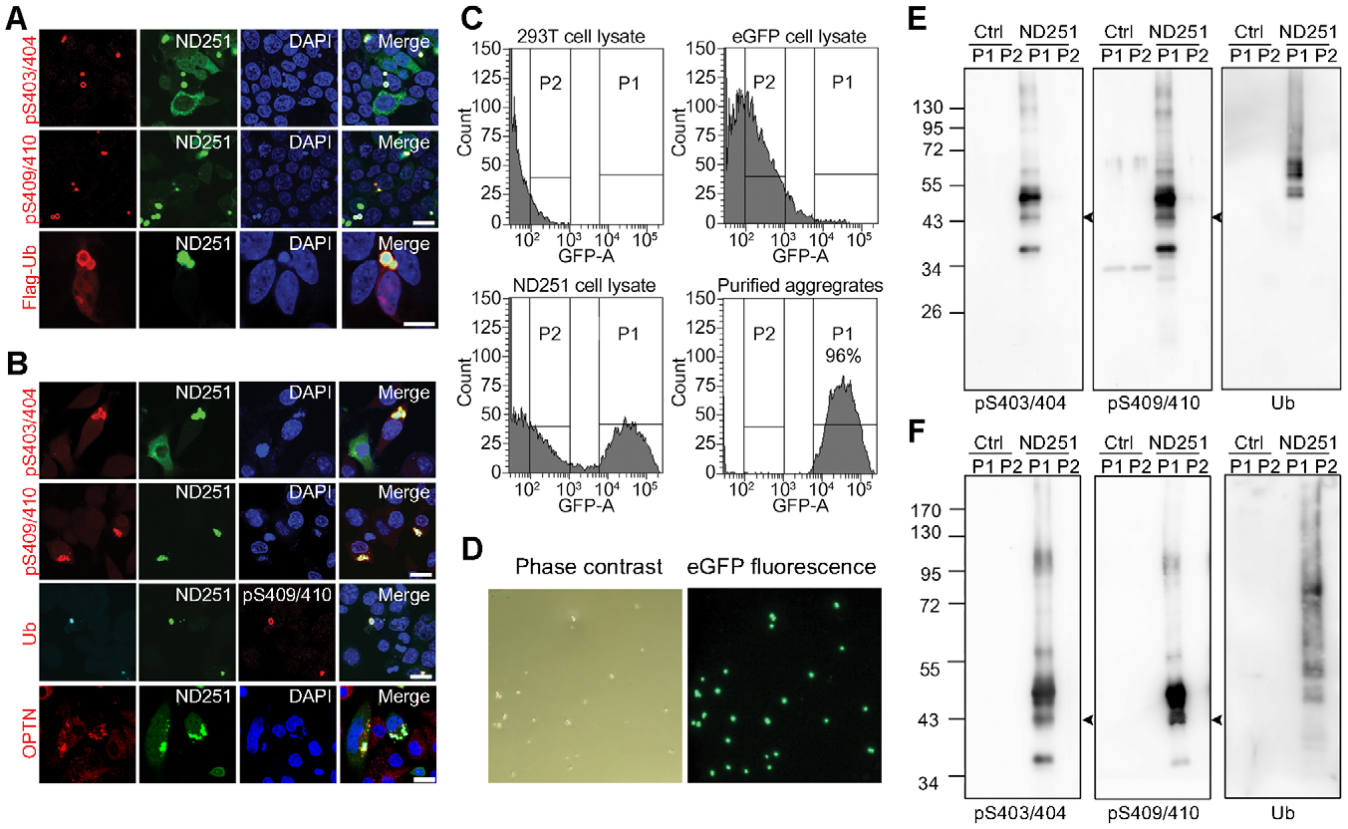
ND251 aggregates recapitulating important features of TDP inclusions in ALS/FTLD-U. Confocal micrographs of HEK293T (co-expressing flag-tagged ubiquitin) (A) and Neuro2a (*B*) cells expressing ND251, stained with anti-pS403/404 or pS409/410 antiserum (red), anti-flag mAb, anti-ubiquitin (Ub) or anti-optineurin (OPTN). Scale bar = 20 µm. (*C*) Flow cytometry profile of the GFP fluorescence signal of cell lysates from 293T cells (left upper), 293T cells expressing eGFP (right upper) and 293T cells expressing ND251 (left lower). Peak 1 (P1) in ND251 lysates was formed by ND251 aggregates. Both fractions were collected by FACSAria cell sorter. Isolated P1 materials of the ND251 samples (right lower) reached >96% of purity. (*D*) Phase contrast (left) and fluorescent (right) micrographs of the isolated P1 particles viewed with Nikon Eclipse TE-2000U fluorescent microscope. All of the visible structures with the phase contrast lens emitted bright green fluorescence, validating their identity as ND251 inclusions. Western blot of collected P1 (aggregates) and P2 (non-aggregates) materials of 293T (*E*) and Neuro2a (F) cells expressing eGFP (Ctrl) or ND251 probed with anti-pS403/404, pS409/410, anti-ubiquitin (Ub) and anti-eGFP antibodies. As noted, the pattern recapitulated that of TDP inclusions in ALS/FTLD-U.

To determine if the TDP aggregates developed in our cell culture system served as useful models for TDP inclusions in ALS/FTLD-U cases, hyperphosphorylation and polyubiquitination were characterized in both non-neuronal cell line HEK293T and a neuroblastoma cell line Neuro2a. Western blot analysis revealed strong signals of phosphorylated Ser^403^/Ser^404^ and Ser^409^/Ser^410^ epitopes in the insoluble fractions of truncated TDP species (Fig. S1). Given the highest aggregation propensity, ND251 was used most in the following studies, with validation using ND207. As shown in Fig. 2A and 2B, ND251 aggregates in 293T and Neuro2a cells contained phosphorylated Ser^403^/Ser^404^ and Ser^409^/Ser^410^ epitopes and were ubiquitinated. Consistently, exogenously expressed flag-tagged ubiquitin (Flag-Ub) was incorporated into the ND251 aggregates (Fig. 2A). Very recently, optineurin protein was shown to colocalize with TDP-positive inclusion bodies in ALS [26]. Interestingly, optineurin also coexisted with ND251 aggregates (Fig. 2B).

To further characterize these inclusions biochemically, aggregated and non-aggregated ND251, represented by the P1 and P2 fraction, respectively, were isolated from transfected cells using FACSAria cell sorter (Fig. 2C and 2D). More than 96% of the isolated P1 materials were in fact aggregates, indicative of high purity of aggregates (right lower panel in Fig. 2C and Fig. 2D). Again, Western blot analysis revealed strong ubiquitin signal with smearing, and phosphorylated Ser^403^/Ser^404^, and Ser^409^/Ser^410^ epitopes in these isolated ND251 aggregates, but weak or no signal in P2 fraction or non-aggregated ND251 or control eGFP samples in HEK293T (Fig. 2E) and Neuro2a cells (Fig. 2F). Taken together, these data demonstrated that ND251 aggregates closely resembled ALS/FTLD-U inclusions not only in the intrinsic properties of TDP elements, but also in their extrinsic properties evidenced by interacting with optineurin, supporting their use as feasible cell models.

### Aggregation propensity of TDP decreased by hyperphosphorylation-mimetic mutations

To investigate the effect of the hyperphosphorylation on the formation of TDP inclusions, we generated fTDP and ND251 mutants with the five serine residues (379, 403, 404, 409 and 410) mutated to either alanine (S5A) (phosphorylation-deficient), aspartic acid (S5D) or glutamic acid (S5E) (phosphorylation-mimetic). The phosphorylation-mimetic properties of these mutants were first characterized by the phospho-specific antisera. As expected, S5D and S5E mutants of fTDP and ND251 were clearly recognized by anti-pS403/404 or anti-pS409/410 antisera, but the S5A mutants were not (Fig. S2). Notably, the S5D and S5E had an apparent molecular weight larger than the fTDP or S5A, an additional feature consistent with hyperphosphorylation form of TDP. These data supported the hyperphosphorylation-mimetic property of S5D and S5E.

To determine if phosphorylation status impacted TDP on its aggregation, we first investigated the aggregation propensity of fTDP vs. the single, double phosphorylation sites mutants as well as S5A, S5D or S5E mutants (Fig. S3A and S3B). fTDP, S5A, S5D or S5E exhibited primarily a diffuse nucleoplasmic pattern with few aggregates ; however, Western blot analysis revealed a mild increase in the amount of S5A partitioned into the urea (U) fraction, when compared with fTDP or S5E or S5D (Fig. S3C). In addition, filter trap assay which is another method widely used to study aggregation propensity showed a decrease in the signal of S5E when compared with that of fTDP (Fig. S3D). These data suggested that hyperphosphorylation status might decrease, rather than increase, the aggregation propensity of fTDP.

**Figure 3:**
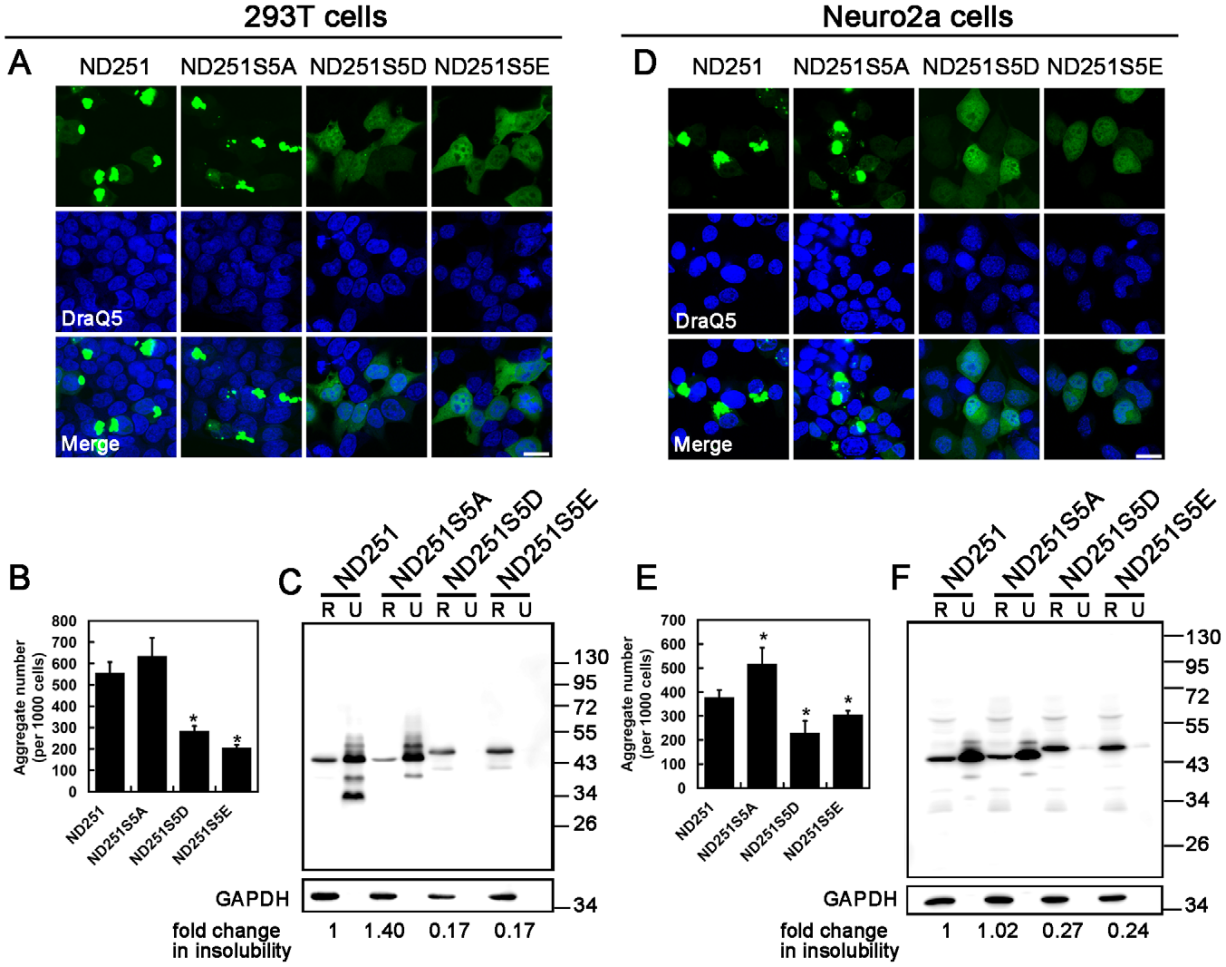
Effects of phosphorylation site mutations on aggregation propensity in 293T (*A–C*) and Neuro2a (*D–F*) cells. Scale bar = 20 m. (*A*) and (*D*): Confocal micrographs of 293T and Neuro2a cells expressing ND251, ND251S5A, ND251S5E or ND251S5D. (*B*) and (*E*): Quantitative analysis of the number of aggregates per 1000 cells. Note, ND251 and ND251S5A readily formed aggregates, but ND251S5E and ND251S5D formed fewer aggregates. (*C*) and (*F*): Western blot analyses of RIPA-solubility of ND251S5A, ND251S5E and ND251S5D normalized to ND251. Anti-GFP antibody was used for the detection of truncated TDP. The fold change was quantified by ImageQuant software.

We next examined the effect of the same hyperphosphorylation site mutations on the aggregation propensity of truncated TDP species, and found these mutations exerted a much more significant impact on the truncated TDP than fTDP. The numbers of aggregates formed by hyperphosphorylation-mimetics ND251S5D and ND251S5E significantly decreased by 50% and 65%, respectively, in HEK293T (Fig. 3A and 3B) and by 50% and 25%, respectively, in Neuro2a cells (Fig. 3D and 3E). In contrast, an increase in the number of aggregates by 15% and 34% (statistically significant) was noted for ND251S5A in HEK293T (Fig. 3B) and Neuro2a cells (Fig. 3E) in comparison with ND251. Consistently, quantitative analyses of the corresponding Western blots showed a decrease in the solubility of the ND251S5A in comparison with ND251; however, ND251S5D and ND251S5E proteins became highly soluble in RIPA buffer in both 293T (Fig. 3C) and Neuro2a (Fig. 3F) cells.

Since the ∼25 kDa cleaved fragment was hyperphosphorylated to a much higher extent than full length TDP in ALS/FTLD-U [38], the same experiments were repeated with ND207 which modeled after this fragment. Similarly, the number of aggregates formed by ND207S5E dramatically decreased by 91% in Neuro2a, and by 56% in HEK293T cells when compared with ND207 (Fig. S4A and S4E; quantified in Fig. S4B and S4F), and the number of aggregates of ND207S5A increased by 13% in Neuro2a, although the number remained comparable to that of ND207 in HEK293T cells. The aggregates formed by ND207S5A decreased by 46% in size and that of ND207S5E inclusions, by 67% in Neuro2a cells (Fig. S4C); however, no significant difference in size was noted for inclusions formed by the ND207, ND207S5A or ND207S5E in 293T cells (Fig. S4G). Western blot analyses showed that RIPA solubility of ND207S5A decreased or remained unchanged in comparison with ND207, but ND207S5E became highly soluble in both cell lines (Fig. S4D and S4H).

To rule out the possibility that the aggregation propensity of truncated forms of TDP might be altered by the large eGFP tag, similar experiments were repeated with myc-tagged truncated TDP (as described in Text S1). Similar to eGFP-ND207, Myc-ND207 also formed ubiquitinated aggregates which colocalized with optineurin in Neuro2a (Fig. S5B) and HEK293T (Fig. S5D) cells. Myc-ND207S5E formed significantly fewer aggregates in both cell lines (Fig. S5A and S5C; quantified in Fig. S5E). In addition, in contrast with Myc-ND207 and Myc-ND207S5A, Myc-ND207S5E was highly soluble in RIPA buffer (Fig. S5F). These data agreed with the results obtained with eGFP-ND207S5E.

Flag-, Myc-, His- and V5-tagged ND251 constructs were also made and tested. Unfortunately, none of these could be expressed in either HEK293T or Neuro2a and other cell lines. The carboxyl terminus is predicted to be a disordered region by PONDR [43], lack of expression of these ND251 might be caused by rapid degradation after synthesis. Notably, the myc-tagged ND207 had much higher aggregation propensity than eGFP-tagged counterpart (69.9±1.8% vs. 4.6±0.7%). In addition, the presence of eGFP stabilized the expression of ND251 in comparison with myc tag. These results showed that the tags could influence the property of the fused segment of TDP to some extent. In spite of this, our data supported the notion that hyperphosphorylation negatively impacted TDP on its ability to form inclusions.

### Additive effects of individual phosphorylation site mutations on aggregation propensity

To investigate the effect of phosphorylation status of these five serine residues individually or in combination, a series of phosphorylation mutant constructs were generated and examined. As shown in Fig. S6A and S6B, the single mutant ND251S379A, and S403/404A and S409/410A double mutants showed no obvious effect on the number of inclusions. In contrast, ND251S379E and double mutants (S403/404E and S409/410E) showed an 18% and ∼40% reduction in the number of inclusions, respectively. The number of inclusions formed by S379E/S403/404E and S379E/S409/410E triple mutants further reduced to ∼50%, and S403/404/409/410E quadruple mutant, to 38% (Fig. S6C and S6D). The corresponding Western blot analyses also showed a positive correlation between the number of serines mutated to alanines and the amount of the insoluble proteins in the urea fraction, and an inverse relationship when serines were mutated to glutamic acids (Fig. S6E). These data demonstrated that phosphorylation status of all five serine residues participated in modulation of aggregation propensity of ND251, and had an additive effect on the final outcome.

### Higher solubility of hyperphosphorylated ND251 than that of unphosphorylated ND251

**Figure 4:**
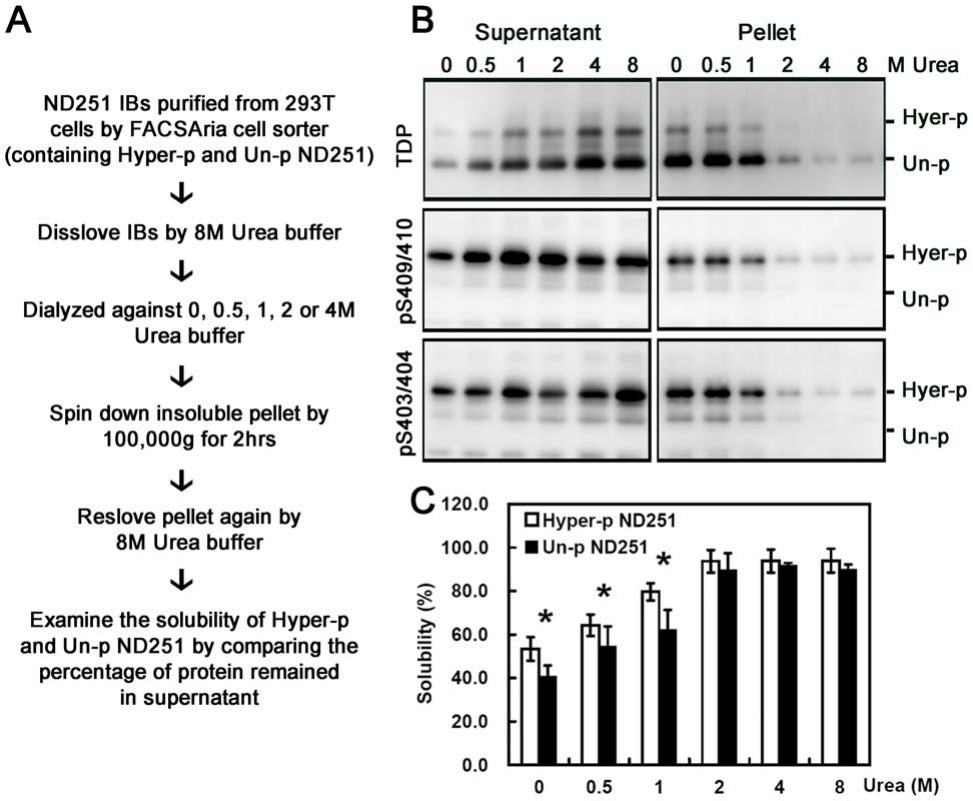
Hyperphosphorylated ND251 had higher solubility than unphosphorylated form in vitro. (*A*) Experimental procedures for analyzing the solubilities of hyper- (Hyper-p) and unphosphorlylated (Un-p) ND251 from ND251 aggregates isolated from HEK293T cells. (*B*) Representative Western blot probed with homemade TDP, pS403/404 and pS409/410 antiserum to determine solubility of hyperphosphorylated and unphosphorylated ND251 in buffers with urea concentrations as indicated. The solubility was determined by level of protein partitioned into supernatant or pellet after dialysis against 0, 0.5, 1, 2 or 4 M Urea buffer. (*C*) Quantitative results on solubility of Hyper-p and Un-p ND251. Results were quantified by ImageQuant software from quadruplicate experiments. The solubility was represented by the following formula: (intensity of protein in supernatant)/(sum of the intensity of protein in supernatant and pellet).

To address the issue of hyperphosphorylation and aggregation propensity without complications from artificial mutations, we decided to isolate the ND251 aggregates and to compare in various buffers the solubility of the hyperphosphorylated species with that of unphosphorylated species from the isolated aggregates (Fig. 4A). As shown in Fig. 4B and 4C, hyperphosphorylated form indeed had higher solubility than unphosphorylated form in buffers containing 0–1 M urea. The difference in the solubility between these two disappeared in the presence of 2 M urea or more. These results further supported that the solubility of truncated TDP increased with hyperphosphorylation.

### The effect of Casein kinase 2 on the formation of TDP inclusions

**Figure 5:**
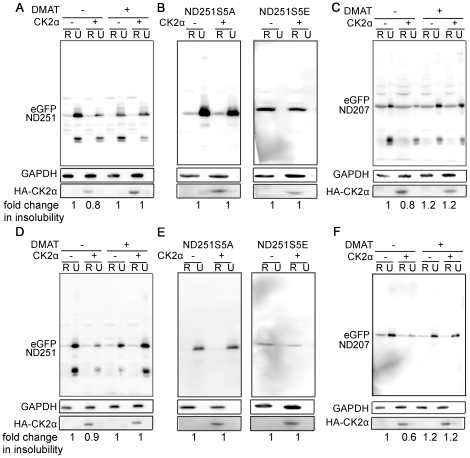
The effect of CK2α on the aggregation propensity of truncated TDP. Exogenous expression of CK2α was shown with anti-HA (HA) mAb. Expression of CK2α decreased the insoluble fraction of ND251 in both 293T (*A*) and Neuro2a (*D*) cells, and CK2α-specific inhibitor DMAT (20 µM for 24 hr) treatment blocked this effect. Similar results were also obtained with ND207 in 293T (C) and Neuro2a (F) cells. In contrast, CK2α either slightly decreased or failed to change ND251S5A solubility in 293T(*B*) and Neuro2a (*E*) cells, and showed no effect on ND251S5E (*B,D*). GAPDH was used as loading control. The fold change was quantified by ImageQuant software by comparing the insolubility to ND207 or ND251 relatively.

Casein kinases (CKs) 1 and 2 were reported to phosphorylate these five serines, and promoted the ability of TDP recombinant protein to form filaments *in vitro*. We first examined if fTDP or truncated TDP altered endogenous CK2 activity by *in vitro* kinase assay (as described in Text S1). As shown in Fig. S7, neither fTDP nor ND207 or ND251 overexpression changed endogenous CK2 activity in neuro2a cells. Next, we expressed CK2α and examined its effect on aggregation propensity of truncated TDP. As shown, expression of CK2α increased phosphorylation status of 403/404 and 409/410 serine residues (Fig. S8), but decreased the amount of insoluble ND251 in the urea fraction in both 293T and Neuro2a cells (Fig. 5A and 5D). This effect could be blocked with DMAT, a CK2-specific inhibitor. A similar result was also obtained with the ND207 (Fig. 5C and 5F). To further assess if CK2α-regulated solubility changes involved phosphorylation of these serine residues, experiments were repeated with ND251S5A and ND251S5E. Although the solubility of ND251S5A slightly increased with expression of CK2α in 293T cells, but the change was less than that of ND251. No change was observed in Neuro2a cells (Fig. 5B). More importantly, ND251S5E remained highly soluble with or without CK2α in either 293T or Neuro2a cells (Fig. 5E). These data demonstrated that exogenous expression of CK2α enhanced the solubility of truncated TDP by regulating the phosphorylation status of these serine residues.

### Cytotoxic effects of ND251 or ND207 aggregates enhanced by S5A mutation, but alleviated by S5E mutation

**Figure 6:**
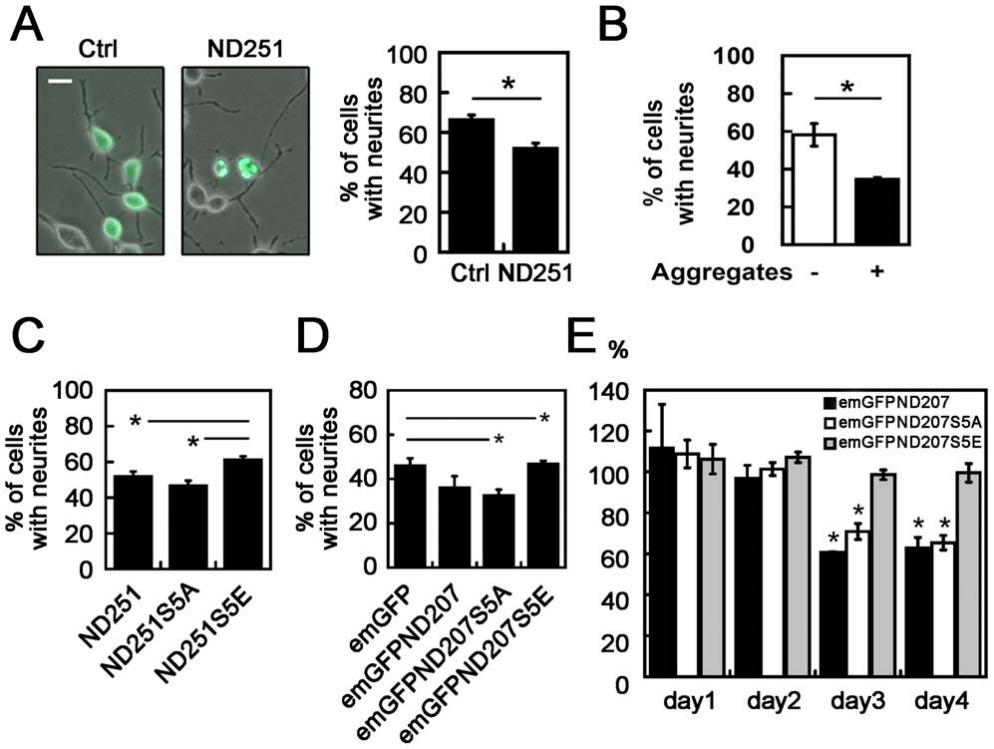
Adverse effect of ND251 aggregates on neurite outgrowth. (*A*) *Left:* Merged phase contrast and GFP fluorescent micrographs of Neuro2a cells which expressed eGFP (Ctrl) or ND251 and were induced with 10 µM retinoic acid for 48 hrs. The neurites were defined as cellular processes 2 ≧times of diameter of cell body. Cell with neurites were quantified by Metamorph software. Neurites were readily noted in Ctrl cells, but absent in two cells with ND251 aggregates. Scale bar = 10 µm. *Right:* Quantitative bar graph on percentage of Neuro2a cells with extended neuritis. Note: ND251 caused a decrease in neurite-bearing cells compared with Ctrl eGFP cells. (*B*) Quantitative results of neurite-bearing cells in ND251-expressing Neuro2a cells with or without aggregates. As shown, Neuro2a cells with aggregates had 50% less of cells with neurites than those without aggregates. (*C*) Percentage of Neuro2a cells with neurites for cells expressing ND251, ND251S5A and ND251S5E. Cells expressing ND251S5A had a further decrease in the percentage of neurite-bearing cells in comparison with ND251. In contrast, the percentage for ND251S5E was higher than that of ND251 and the percentage reverted to the level comparable to that of the Ctrl in Fig. 6B. (*D*) Percentage of Neuro2a cells with extended neurites for cells expressing inducible emGFP, emGFPND207, emGFPND207S5A and emGFPND207S5E. Cells expressing emGFPND207 and emGFPND207S5A had fewer neurite-bearing cells in comparison with emGFP (Ctrl) group. In contrast, the percentage for ND207S5E was higher than that of ND207 and the percentage reverted to the level comparable to that of the Ctrl group. (*E*) MTT assay for neuro2a cells expressing inducible emGFP, emGFPND207, emGFPND207S5A and emGFPND207S5E. Percentages of cell growth rated to emGFP were compared for 4 days. emGFPND207 and emGFPND207S5A inhibited ∼40% growth of neuro2a cells at day3 and day4. On the contrary, cell growth of emGFPND207S5E expressing cells is similar to that of emGFP expressing cells. *, P<0.05.

To explore the functional significance of hyperphosphorylation on TDP aggregates, 2 different cell assays were tested. The first was retinoic acid (RA)-induced neurite outgrowth of Neuro2a cells. Since the undifferentiated cells contained short processes, the neurites were defined as processes at least twice longer than that of cell bodies. Our data showed that ND251 aggregates exerted inhibitory effect on neurite outgrowth. Overall, percentage of Neuro2a cells bearing extended neurites decreased from 66% in eGFP control cells to 52% in cells expressing ND251 after 48 hrs RA treatment (Fig. 6A). This negative impact appeared mild because only a small percentage (13.1%±1.7%) of transfected Neuro2a cells actually developed aggregates. To address this issue more precisely, the ND251-expressing cells were separated into 2 groups: without aggregates and with aggregates. The percentage of neurite-bearing cells was then separately calculated in each group. Notably, the latter group with ND251 aggregates had a significant drop in neurite outgrowth by 44% when compared with the former group without aggregates (Fig. 6B). These results revealed a cytotoxic effect of ND251 aggregates.

To examine the influence of the hyperphosphorylation on the cytotoxicity of ND251 aggregates, neurite outgrowth was studied in cells expressing ND251 and its mutants. As shown in Fig. 6C, the overall percentage of Neuro2a cells with neurites further decreased from 52% in cells expressing ND251 to 47% in cells expressing ND251S5A, but increased to 62% in ND251S5E-expressing cells, comparable to that in eGFP control cells (Fig. 6A).

Similar results were obtained in cells expressing emGFPND207 and its mutants. The tet-off system was used to express emGFPND207 and its mutants because more aggregates could be induced. As shown in Fig. 6D, cells expressing emGFPND207 and emGFPND207S5A had lower percentage of neurite-bearing cells in comparison with emGFP control; in contrast, cells expressing emGFPND207S5E reverted the percentage back to the level comparable to that of the emGFP control group.

The second test was cell growth by MTT assay (Fig. 6E). Again, the emGFPND207 and emGFPND207S5A had ∼40% less neuro2a cells on days 3 and 4. On the contrary, growth of emGFPND207S5E expressing cells remained at a similar pace to that of emGFP expressing cells. Taken together, these results demonstrated that truncated TDP aggregates exerted cytotoxic effect, which was alleviated by hyperphosphorylation-mimetic mutations.

### Phosphorylation and ubiquitination occurring after aggregation

**Figure 7:**
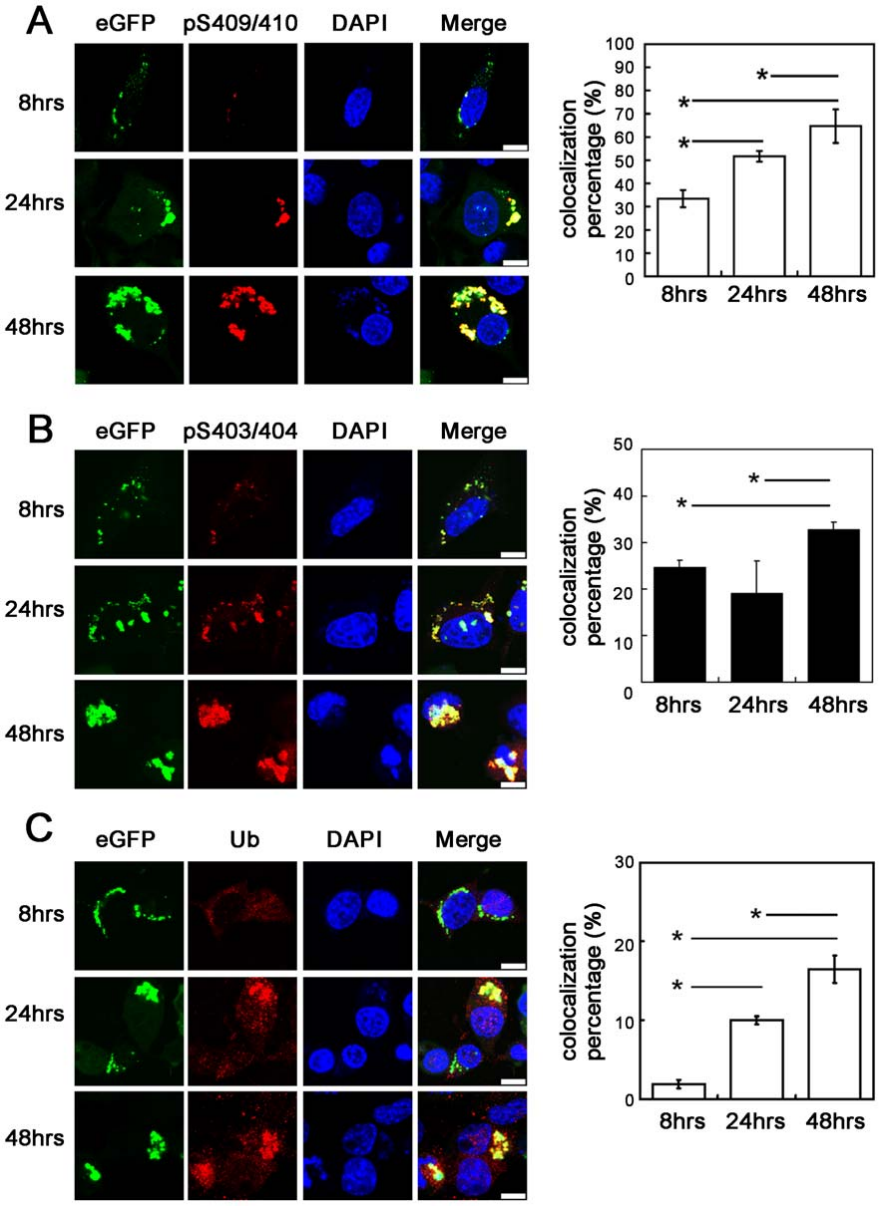
Phosphorylation and ubiquitination of ND251 aggregates increased with time. Confocal micrographs of Neuro2a cells expressing ND251 were stained with (*A*) anti-pS409/410, (*B*) anti-pS403/404 and (*C*) anti-Ubiquitin antiserums. Scale bar = 10 µm. ND251 aggregates with phosphorylated 409/410, phosphorylated 403/404 or ubiquitinated epitopes at different time point were calculated by MetaMorph software. *, P<0.05.

Previous studies [38], [44] suggested hyperphosphorylation as a precursor change in favor of formation of TDP inclusions. To clarify this important issue, we investigated the temporal sequence between aggregation and phosphorylation as well as ubiquitination using our cell culture model. As shown in Video S1, Fig. 7A and 7B, ND251 aggregates started as multiple small punctate structures distributed throughout the cytoplasm. With time, small aggregates gained in size by self-growth and merging with other aggregates, and eventually formed large inclusions in fewer numbers. The majority of ND251 aggregates were phosphorylated 48 hours after transfection, but a certain percentage of aggregates, particularly smaller ones, were not labeled with either anti-pS403/404 or anti-pS409/410 antiserum. To examine in more details, quantitative analysis was done in three different time points, which revealed that the percentage of ND251 aggregates with phosphorylated Ser^409/410^ signal increased from 33.5±3.7% at 8 hrs to 51.6±2.3% at 24 hrs, and to 64.6±7.2% at 48 hrs (Fig. 7A). The percentage of ND251 aggregates with phosphorylated Ser^403/404^ signal also increased from 24.7±1.38% at 8 hrs to 32.8±1.63% at 48 hrs (Fig. 7B). Notably, the ubiquitination of ND251 aggregates followed a similar trend, and increased from 1.90±0.55% at 8 hrs to 10±0.51% at 24 hrs, and 16.5±1.73% at 48 hrs (Fig. 7C). It was apparent that the aggregates which were not phosphorylated were primarily small punctate structures, regardless of the time point. These data indicated that phosphorylation and ubiquitination occurred after the formation of aggregates.

### Validation of the effect of hyperphosphorylation site mutations on aggregation propensity in transgenic models of *Drosophila melanogaster*

**Figure 8:**
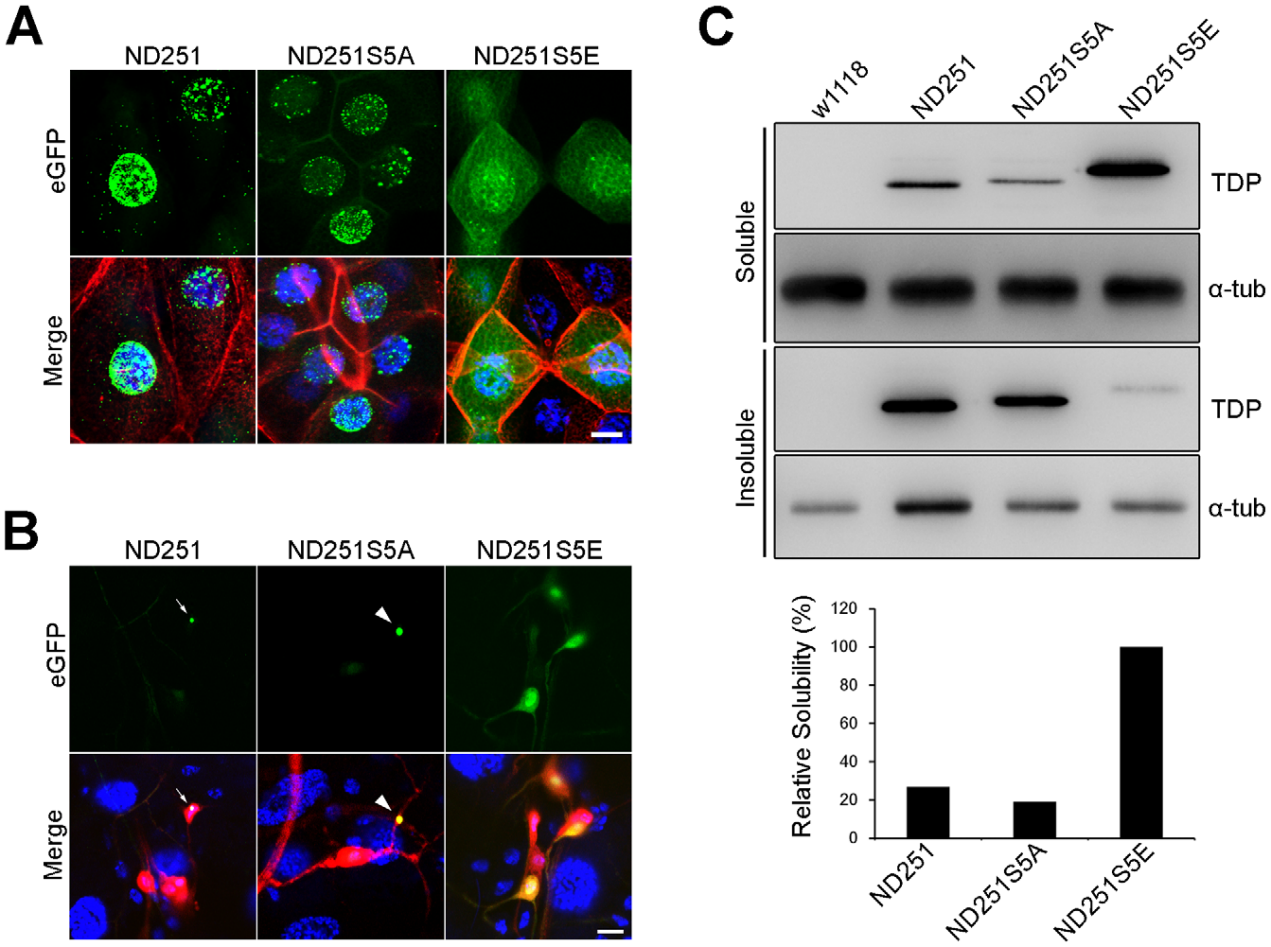
Aggregation propensity of ND251, ND251S5A and ND251S5E in transgenic Drosophila model. (A) Confocal micrograph of aggregates formed by ND251 and ND251S5A in the salivary gland of third instar larvae. Hyperphosphorylation-mimetic ND251S5E appeared diffusely distributed in the nuclei and cytoplasm. Green: ND251 variants, red: phalloidin, blue: DAPI. (B) ND251 or ND251S5A aggregates in the soma (arrow) and dendrite (arrowhead) of dda neurons. In contrast, ND251S5E was diffusely distributed in dendrite and axon. Green: ND251 variants tagged with GFP, red: DsRed revealing neurite structures, blue: DAPI. (C) Western blot analysis of solubility of ND251, ND251S5A and ND251S5E *in vivo* As shown, solubility of ND251S5E was much higher than the other two. Adult heads expressing ND251 variants were collected and extracted in RIPA buffer. Relative solubility was performed by comparing the normalized densitometry of each to ND251S5E. Scale bar, 25 µm (A), 10 µm (B).

To investigate if the hyperphosphorylation exerted a similar inhibitory effect on the aggregation propensity of N-terminal truncated TDP *in vivo*, we generated *UAS-ND251*, *UAS-ND251S5A* and *UAS-ND251S5E* transgenic *Drosophila melanogaster*, and crossed them with *ywhsFLP*; *Actin-Gal4>y+>UAS-β-galactosidase* flies. Salivary gland was examined first because of its giant epithelial cells with easily distinguishable cytoplasm and nucleus. As shown in Fig. 8A, after heat shock at 37°C, the ND251 and ND251S5A formed a number of aggregates distributed within nuclear and cytoplasmic compartments. In contrast, ND251S5E was diffusely distributed in both compartments with few aggregates observed.

To examine the aggregation propensity of these proteins in the fly nervous system, we then crossed the transgenic flies with pan-neuronal GAL4 flies (*UAS-DsRed*; *elav-Gal4/+*). For reasons not yet clear, expressions of ND251 and ND251S5A were lower and more restricted than that of ND251S5E across all different lines (data not shown). Despite that, both ND251 and ND251S5A were found to form aggregates in perikarya (Fig. 8B, arrow) and dendrites (Fig. 8B, arrowhead) of multi-dendritic dendrite-arborization (md-da) neurons [45]. Most of the aggregates were located in the cytoplasmic/dendritic compartments, and few were observed in nuclei. Interestingly, our fly models might be the first to develop TDP aggregates in dendrites. In contrast, hyperphosphorylation-mimetic ND251S5E showed a pattern of diffuse distribution in nuclei, soma, and neurites (Fig. 8B).

Consistent with morphologic observations, the corresponding Western blot analysis of the solubility revealed that ND251 and ND251S5A extracted from heads of the adult crossed transgenic flies were much less soluble than ND251S5E (Fig. 8C). Taken together, our *in vivo* data also supported the notion that hyperphosphorylation reduced the aggregation propensity of ND251.

## Discussion

In this study, we have established and used cell and drosophila models to specifically address the effect of hyperphosphorylation on the formation of TDP inclusions. Both models revealed similar conclusions that hyperphosphorylation reduced aggregation propensity of TDP. Our studies were conducted with ND251 and ND207 truncated TDP. The ND207 simulated the 25 kDa cleaved TDP fragment of FTLD-U/ALS. A recent study found a TDP fragment truncated at glutamic acid 246 in FTLD-U brains, which differed from ND251 by only 5 amino acids [46]. This finding lends support to the pathophysiological relevance of ND251. Furthermore, ND251 and ND207 aggregates shared similar properties in our study and recapitulated pivotal features of diseased TDP inclusions, such as hyperphosphorylation, polyubiquitination and colocalization with optineurin. Therefore, our models might well serve as feasible tools to probe into some essential issues relevant to TDP pathology.

TDP undergoes several pathognomonic changes in ALS/FTLD-U, such as aberrant cytoplasmic translocation, 25 kDa carboxyl terminal fragment by putative protease cleavage and hyperphosphorylation. Several recent studies were designed to elucidate the role of the pathognomonic changes of TDP in regard to formation of TDP inclusions and diseases. For instance, cytoplasmic translocation and putative protease cleavage were shown in these studies as important precursor changes for TDP inclusions [32], [33], [34], [35], [47], [48]. Consistent with this, we also found that TDP deleted of 104 amino acids and more from amino terminus exhibited higher propensity for aggregation than full length TDP. ND251 was primarily composed of the carboxyl terminus, and had the highest aggregation propensity among the three truncated forms studied here. Our recent study showed that the D1 peptide derived from the glycine-rich domain of TDP indeed readily formed fibrils [43]. Therefore, the carboxyl terminus is likely the region responsible for self-aggregation in ALS/FTLD-U. TDP is known to interact with multiple proteins involved in mRNA processing, translation and microRNA biogenesis [49], [50]; however, these interactions appear to be lost when TDP forms aggregates [51]. The carboxyl terminus is predicted to be a disordered region by PONDR [43], consistent with its reported role in protein interactions [52], [53], [54]. In fact, TDP in cells and animals is highly soluble, but recombinant TDP protein is very prone to aggregate (data not shown). These data strongly indicate that protein interactions help prevent TDP from aggregation in cells. Notably, the great majority of >30 mutations in gene *TARDBP* in familial ALS occur within the carboxyl terminal region. It is probable that mutations may lead to aggregation by disrupting physiological protein interactions. The role of amino terminus needs further study, but it may help TDP maintain normal structure and interaction with other proteins. This notion provides a rational model to explain why protease cleavage/truncation increases formation of TDP inclusions.

Concerning hyperphosphorylation, the seminal discovery by Hasegawa et al. [38], and several subsequent studies [37], [39], [55] have firmly established a tight link between the inclusions of ALS/FTLD-U and hyperphosphorylation at serine379, serine403, serine404, serine409 and serine410. However, the cause-effect relationship between these two remains to be described. In the same study, Dr. Hasegawa showed that recombinant CKs was capable of phosphorylating these serine residues and promoting fibrillation of full length TDP *in vitro* [36], [38]. Their findings suggested that, as cytoplasmic translocation and protease cleavage, hyperphosphorylation also contributed to formation of TDP inclusions. Therefore, we were surprised by the results that hyperphosphorylation-mimetic mutations in fact decreased the aggregation propensity of the truncated TDP (ND251 and ND207) and probably fTDP as well. Given our data that differently tagged truncated TDP and their mutants behaved similarly, the possibility that these results were an artifact induced by the tags could be reasonably ruled out. The discrepancy between our results and previous studies may well lie in different systems that were used. Dr. Hasegawa's study was done with recombinant protein *in vitro*, but our study was conducted in cell and drosophila models. The property of TDP is different *in vivo* and *in vitro* for lack of interacting proteins. Interestingly, during the course of our study, Dr. Hu and colleagues showed that the aggregation propensity of the C-terminal 25 kDa and 15 kDa fragments of TDP was significantly reduced by phospho-mimetic mutations at serine 409 and 410 residues [56], which supported the concept that phosphorylation decreased aggregation propensity of TDP fragments.

Dr. Braak [44] identified novel small dash-like aggregates in ALS neurons using the pS409/410 antiserum. Given their phosphorylated, but ubiquitin- and p62-negative features, these aggregates were interpreted as early lesions which resulted in TDP inclusions, in line with Dr. Hasegawa's proposal. However, this study was conducted with phospho-specific antiserum, the possibility that an existence of earlier unphosphorylated lesions has not been formally ruled out. Earlier, Mori et al [57] discerned three morphologically distinct forms of TDP “pre-inclusions” in ALS, namely linear wisps, dot-like inclusions, and granular structures, and suggested these pre-inclusions matured into skein-like inclusions, speculated round inclusions and large granular aggregates, respectively. But the phosphorylation status of these inclusions remains to be determined. Our live cell imaging study showed that ND251 aggregates started as numerous tiny structures and gained in size by continuous growth and/or fusion with others to form a few or single large aggregates. It is interesting to note that Dr. Braak's dash-like structures and Mori's pre-inclusions are numerous, and the mature large inclusions are few or single in number. Therefore, ND251 aggregates are believed to follow a maturation process similar to that proposed by Mori et al, and serve as a good model for us to sort this out.

One of the keys to deciphering the role of hyperphosphorylation in regard to aggregation is to determine the temporal sequences between these two changes. To directly address this issue, we calculated the percentage of phosphorylated ND251 aggregates as a function of time in our cell models. As we expected, the percentage of phosphorylated ND251 increased with time; however, there were always a certain percentage of inclusions which were not phosphorylated. The phosphorylated ND251 aggregates were larger than unphosphorylated counterparts regardless of time points. These data indicated that aggregation occurred before phosphorylation, and the small unphosphorylated aggregates were likely newly formed aggregate throughout the study course. This interpretation is consistent with the fact that ND251S5A formed aggregates despite its lack of these phosphorylation sites, and ND251S5E had much lower capability to form aggregates. Taken together, we propose that hyperphosphorylation functions as an early, if not the first line, compensatory defense mechanism induced by the formation of TDP aggregates to counteract the process of further aggregation or, alternatively, to dissolve the pre-existing aggregates.

**Figure 9:**
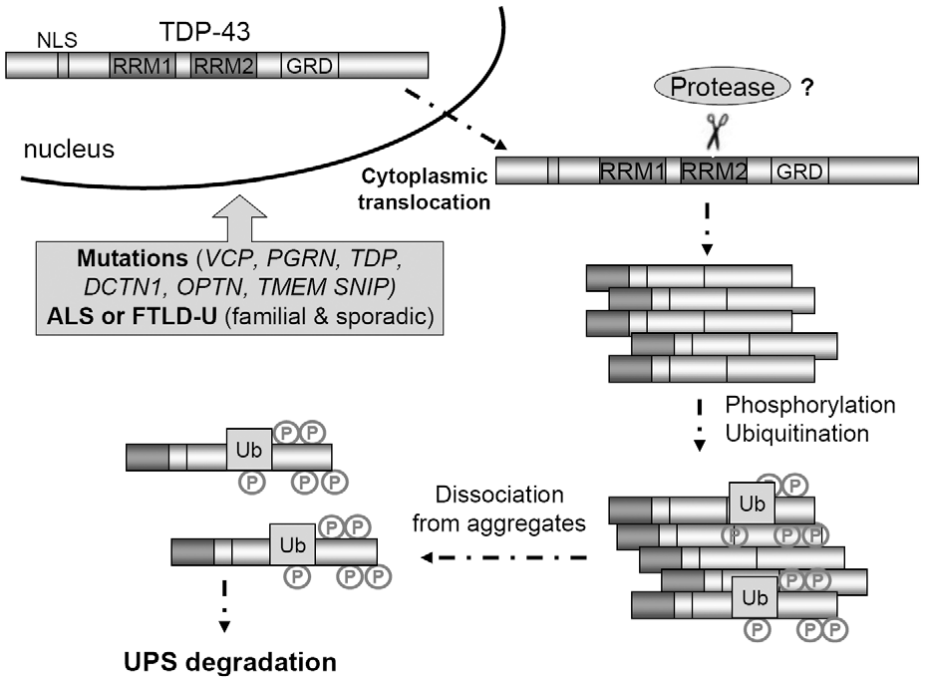
The schematic model of TDP-43 aggregation. Hyperphosphorylation of TDP-43 C-terminal fragments facilitates its disassociation from aggregation which may result in promoting its degradation by ubiquitin proteasome system (UPS).

One of the subsequent key questions then is to address the fate of the hyperphosphorylated TDP species. Although it remains to be tested, a recent interesting study by Dr. Petrucelli and colleagues [58] which showed that TDP aggregates were degraded through proteasome-ubiquitin system (UPS) may have provided an answer. In that paper, they showed that 1) knocking down the heat shock proteins (HSPs) 70 and 90 preferentially increased the phosphorylated species of TDP over the total TDP; 2) degradation of phosphorylated species of TDP was slower than total pool. Although these data were interpreted as evidences supporting the role of hyperphosphorylation in favor of formation of TDP aggregation, they may in fact be more consistent with an alternative view (in concert with our data) that predicts a constant dynamic conversion of unphosphorylated into hyperphosphorylated species which are subsequently subject to the UPS degradation (Fig. 9). From this perspective, the slower degradation curve of phosphorylated TDP may be accounted for by the continuous flow of the unphosphorylated pool into the hyperphosphorylated pool; this view may well explain the preferential increase in the phosphorylated species after HSP knockdown. It will be interesting to determine if hyperphosphorylation functions as a signal for ubiquitination and/or subsequent activation of heat shock/polyubiquitination/protein degradation systems.

Previous studies demonstrated phosphorylation of the five serine residues in ALS/FTLD-U inclusions. However, it is difficult to determine the effect of individual phosphorylated residues on the formation of inclusions. By mutating these residues singly or in combinations, we showed that each individual serine residues contributed to the total capability to modulate the aggregation propensity in a cooperative, not antagonistic, fashion, and these five serines were the key residues to modulate TDP aggregation propensity, given the fact that S5E or S5D mutations exerted a strong inhibition on aggregation. However, TDP aggregates exist in ALS-FTLD-U in spite of hyperphosphorylation. One possibility is that hyperphosphorylated species accounts for a small percentage of aggregated TDP. This was actually shown in previous studies [3], [4]. Alternatively, all of these five serine residues are not phosphorylated on one individual TDP protein. Our data showed that partial phosphorylation achieved only partial protection against aggregation. Therefore, it will be very insightful to precisely determine the extent of phosphorylation of each TDP molecule, and the relative abundance of TDP with one, two, three, four or five phosphorylated serine residues in FTLD-U/ALS inclusions.

## Supporting Information

Figure S1 **Western blot analysis of the phosphorylation status of full length (fTDP) and various truncated forms of TDP protein.** The Western blots were probed with rabbit anti-TDP (top), anti-pSer403/404 (middle) or anti-pSer409/410 (bottom) anti-serum, respectively. As shown, the fTDP was RIPA-soluble (R), and only a small amount was observed in insoluble urea (U) fraction. In contrast, an increase in the amount of TDP partitioned into urea (U) fraction was observed in all three truncated TDP samples (top). The arrow indicated endogenous fTDP protein. The insoluble fractions of all the truncated TDP samples were strongly stained by anti-pSer403/404 or anti-pSer409/410 anti-sera, suggestive of hyperphosphorylation of these aggregated TDP species.
(TIF)

Figure S2 **The phosphorylation-mimetic properties of S5D and S5E mutants characterized by the phospho-specific antisera.** (*A*) RIPA-extracts of Neuro2a expressing S5D or S5E mutants of fTDP (left panel) and ND251 (right panel) were examined by anti-GFP or phospho-specific antisera. As expected, both S5D and S5E mutants of fTDP and ND251 were recognized by anti-pS403/404 or anti-pS409/410 antisera, but the S5A mutants were not. (*B*) Confocal micrographs of ND251S5A, ND251S5D and ND251S5E in Neuro2a cells. Only aggregates formed by ND251S5D and ND251S5E were recognized by anti-pS403/404 or anti-pS409/410 antisera, those by ND251S5A were not. These data indicated that the S5D and S5E shared conformations similar to the hyperphosphorylation epitopes. Scale bar = 20 µm.
(TIF)

Figure S3 **Mild change in aggregation propensity of hyperphosphorylation-deficient or phosphorylation-mimetic mutant of fTDP.** (*A*) Fluorescent micrographs of HEK293T cells expressing fTDP or its mutants with serine379, serines403/404 or serines409/410 mutated either to alanine (A) or glutamic acid (E). These mutants shared a diffuse nucleoplasmic pattern with that of fTDP. (*B*) Mutants of full length TDP with all five serine residues mutated either to alanine (S5A), aspartic acid (S5D) or glutamic acid (S5E) also exhibited a diffuse nucleoplasmic pattern. No significant changes in the number of inclusions were observed. Scale bar in (*A*) and (*B*) = 20 µm. (*C*) Western blot analyses of TDP solubility by sequential extracting Neuro2a cells expressing fTDP, S5A, S5D and S5E with RIPA (R) and urea (U) buffers. As shown, all three proteins were highly soluble evidenced by abundant amounts of proteins in R (soluble) fraction. However, an appreciable increase in the U fraction occurred in S5A sample, suggesting a mild increase in the aggregation propensity. GAPDH was used as loading control. Notably, the S5D and S5E migrated more slowly compared with fTDP and S5A, recapitulating the feature of hyperphosphorylated form of TDP in human ALS/FTLD-U. (*D*) Filter trap analyses of 20 µg of Neuro2a lysate expressing eGFP, fTDP, S5A or S5E mutant scanned by Typhoon 9410 (GE) to reveal GFP signal. The signal represented aggregated proteins trapped on the cellulose acetate membrane (Toyo Roshi Kaisha, Japan). Notably, the S5E was less trapped than fTDP and S5A. Since fTDP formed aggregates in a small percentage of cells, this finding indicated that hyperphosphorylation-mimetic mutation rendered fTDP less prone for aggregation.
(TIF)

Figure S4 **The effect of phosphorylation site mutations on aggregation propensity of ND207.** Confocal micrographs of ND207, ND207S5A and ND207S5E in Neuro2a cells (*A*) or HEK293T cells (*E*). Scale bar in (*A*) and (*E*) = 20 µm. ND207S5E not only formed significantly fewer aggregates in both Neuro2a (*B*) and HEK293T (*F*) cells compared with ND207 or ND207S5A, but also significantly impacted the average size of aggregates in Neuro2a cells (*C*). No significant change in the average size of inclusions was noted in HEK293T cells (*G*). The solubility of ND207, ND207S5A and ND207S5E in Neuro2a (*D*) and HEK293T (*H*) cells were examined by sequential extraction with Western blot. Compared with ND207, ND207S5A was either equally or more insoluble, whereas ND207S5E was highly soluble in RIPA buffer. The fold change of insolubility was calculated with intensity measured by ImageQuant software.
(TIF)

Figure S5 **The aggregation propensities of myc tagged ND207, ND207S5A and ND207S5E were similar to those of eGFP-tagged ND207 counterparts.** Confocal micrographs of myc tagged ND207, ND207S5A and ND207S5E immunostained by anti-myc antibody in Neuro2a cells (*A*) or HEK293T cells (*C*). Scale bar = 20 µm. Similar to eGFP tagged ND207, myc-ND207 also readily formed aggregates which colocalized with both ubiquitin and optineurin signals in Neuro2a cells (*B*) and HEK293T cells (*D*). Compared with myc-ND207 or myc-ND207S5A, myc-ND207S5E formed significantly fewer aggregates in HEK293T (*E*) cells agreed with the quantification done by eGFP-ND207S5E. Moreover, the solubility of ND207, ND207S5A and ND207S5E in HEK293T (*H*) cells were examined by sequential extraction with Western blot. Compared with ND207, ND207S5A showed a decrease in the solubility (R) fraction, but an increase in the insoluble (U) fraction; whereas, ND207S5E was highly soluble in RIPA buffer.
(TIF)

Figure S6 **An inverse relationship between the aggregation propensity of ND251 with the number of serine residues mutated to glutamic acids.** Confocal micrograph montages of ND251 with single/double mutations (*A*), and triple/quadruple mutations (*C*) in HEK293T cells. Scale bar in (*A*) and (*C*) = 20 µm. Quantitative analysis (*B* and *D*) revealed a trend of gradual decrease in the numbers of inclusions formed by ND251 with the increase in the number of S→E mutations. However, S→A mutants did not exhibit a significant change in the ability to form inclusions. *, P<0.05; **, P<0.005; #, P<0.0005. (E) Consistently, Western blot analysis of the RIPA solubility of the ND251 mutants revealed a consistent increase in solubility with an increase in the number of S→E mutations. The S→A mutations did not significantly alter the solubility of ND251.
(TIF)

Figure S7 **CK2 activities in neuro2a cells were not altered by truncated TDP-43 series overexpression.** Endogenous CK2 activity was examined by an in vitro kinase assay employing the measurement of [γ-^32^P]ATP incorporation into the CK2-specific substrate peptide RRREEETEEE. Neither full length TDP-43 (fTDP) nor truncated TDP-43 series (ND207 or ND251) overexpression changed CK2 activity in neuro2a cells. c.p.m.: counts per minute.
(TIF)

Figure S8 **An increase in the phosphorylation status of truncated TDP by CK2α.** Expression of CK2α decreased the insoluble ND251 in urea (U) fraction (left panel). However, CK2α increased the phosphorylation status at both serine 403/404 and serine 409/410 residues of the insoluble ND251 in HEK293T cells by ∼2.64 and 1.9 folds, respectively, after normalization. These data supported the notion that hyperphosphorylation decreased the aggregation propensity of ND251. GAPDH was used as loading control and exogenous expression of CK2α was shown with anti-HA (HA) mAb.
(TIF)

Text S1 **Supplemental methods.**
(DOC)

Video S1 **Live cell image of ND251 aggregates in Neuro2a cells for 24 hrs.** eGFP tagged ND251 were transfected into Neuro2a cells grown in chamber for live cell imaging. After transfection for 4 hrs, images were captured by Axiovert 200 M inverted microscopes (Zeiss) with 40× oil-immersion objectives. Both phase and fluorescence images were captured every 30 minutes for 24 hrs. MetaMorph software (Molecular Devices) was used for data recording and processing.
(AVI)
