# Supplementary figures and images for: Hyperphosphorylation as a Defense Mechanism to Reduce TDP-43 Aggregation
Source: PLoS One. 2011 Aug 5;6(8):e23075. doi: 10.1371/journal.pone.0023075 (PMC3151276; doi:10.1371/journal.pone.0023075)

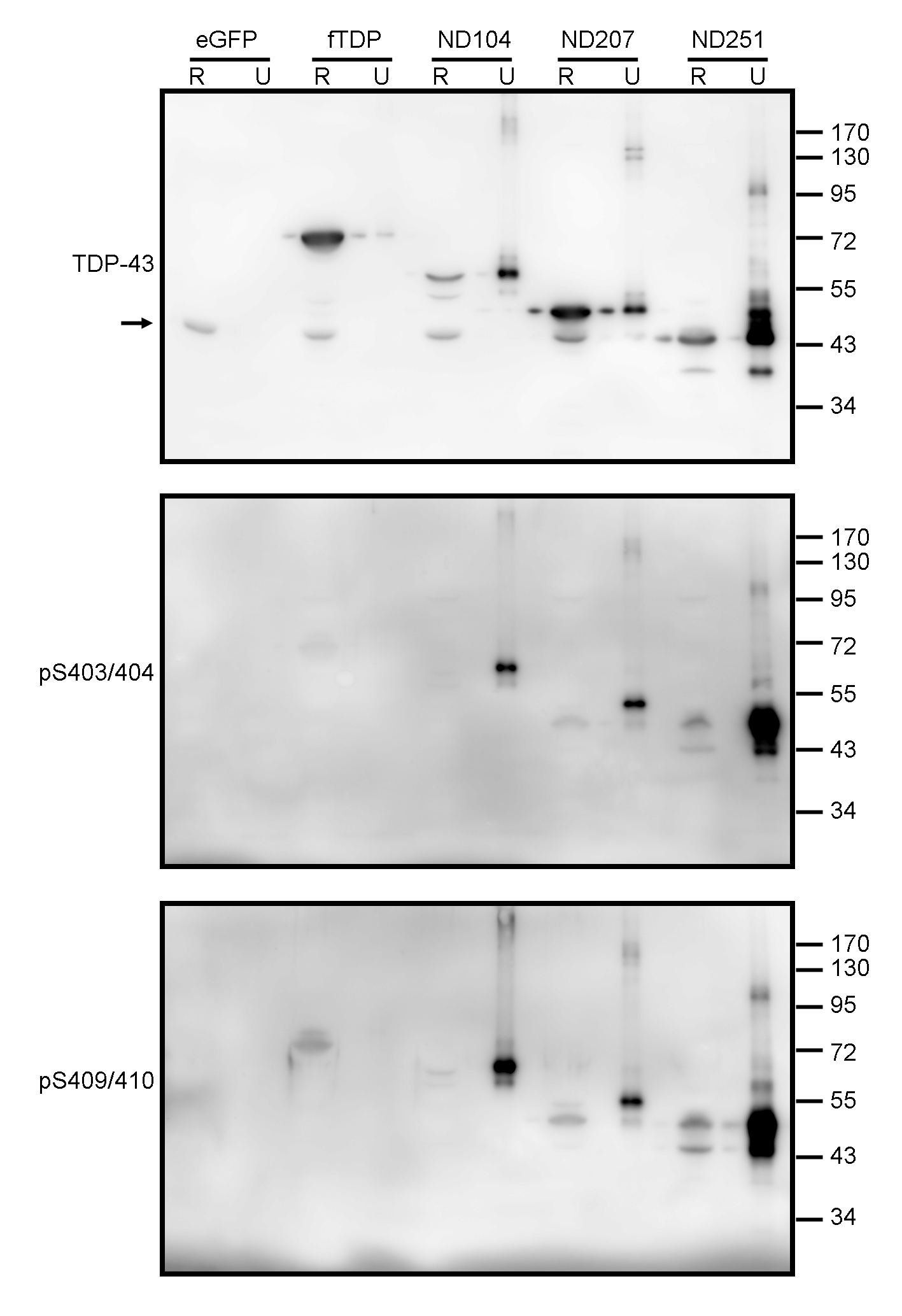

Supplement: Figure S1 — Western blot analysis of the phosphorylation status of full length (fTDP) and various truncated forms of TDP protein. The Western blots were probed with rabbit anti-TDP (top), anti-pSer403/404 (middle) or anti-pSer409/410 (bottom) anti-serum, respectively. As shown, the fTDP was RIPA-soluble (R), and only a small amount was observed in insoluble urea (U) fraction. In contrast, an increase in the amount of TDP partitioned into urea (U) fraction was observed in all three truncated TDP samples (top). The arrow indicated endogenous fTDP protein. The insoluble fractions of all the truncated TDP samples were strongly stained by anti-pSer403/404 or anti-pSer409/410 anti-sera, suggestive of hyperphosphorylation of these aggregated TDP species. (TIF) [file pone.0023075.s001.tif]

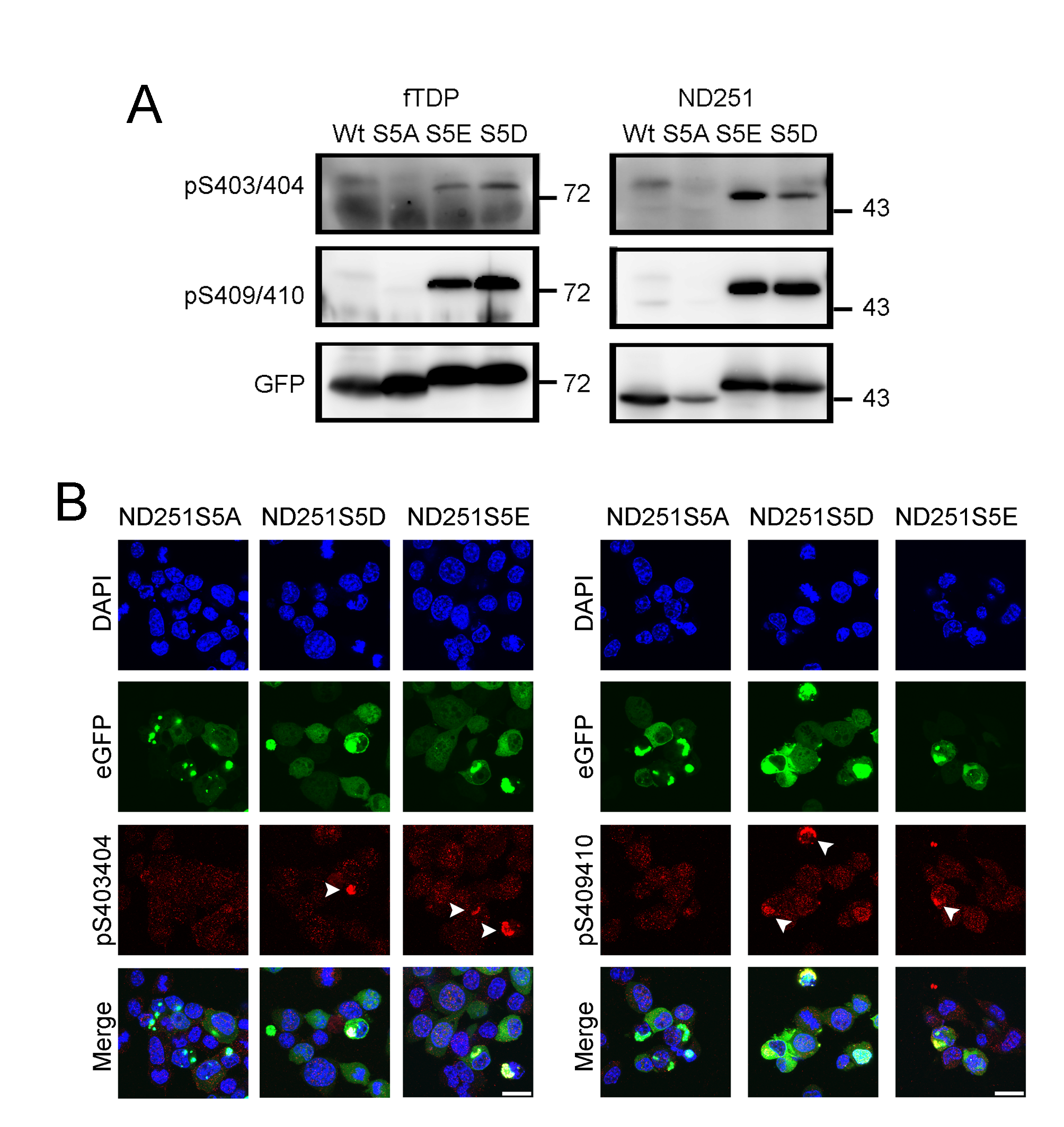

Supplement: Figure S2 — The phosphorylation-mimetic properties of S5D and S5E mutants characterized by the phospho-specific antisera. (A) RIPA-extracts of Neuro2a expressing S5D or S5E mutants of fTDP (left panel) and ND251 (right panel) were examined by anti-GFP or phospho-specific antisera. As expected, both S5D and S5E mutants of fTDP and ND251 were recognized by anti-pS403/404 or anti-pS409/410 antisera, but the S5A mutants were not. (B) Confocal micrographs of ND251S5A, ND251S5D and ND251S5E in Neuro2a cells. Only aggregates formed by ND251S5D and ND251S5E were recognized by anti-pS403/404 or anti-pS409/410 antisera, those by ND251S5A were not. These data indicated that the S5D and S5E shared conformations similar to the hyperphosphorylation epitopes. Scale bar = 20 µm. (TIF) [file pone.0023075.s002.tif]

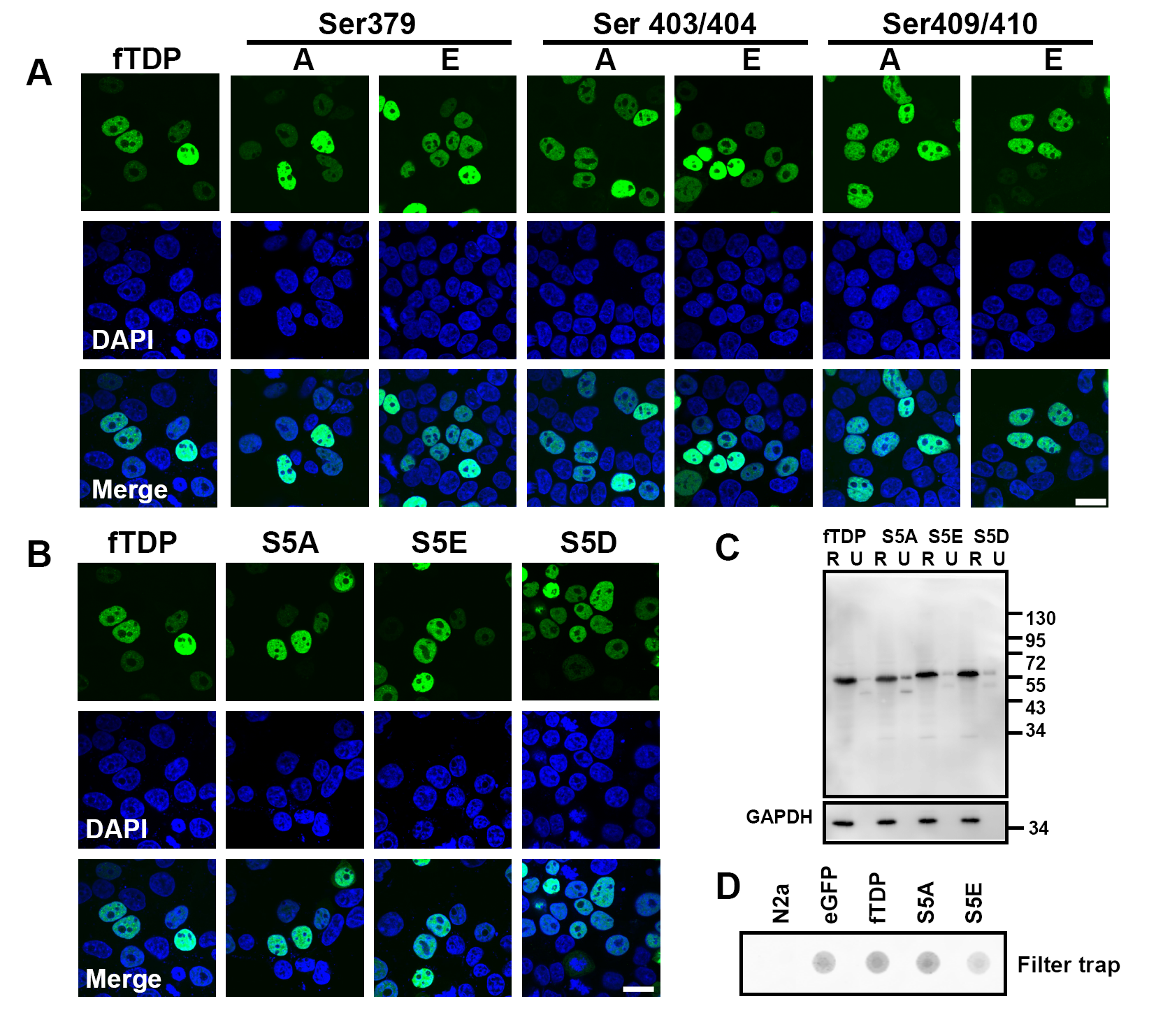

Supplement: Figure S3 — Mild change in aggregation propensity of hyperphosphorylation-deficient or phosphorylation-mimetic mutant of fTDP. (A) Fluorescent micrographs of HEK293T cells expressing fTDP or its mutants with serine379, serines403/404 or serines409/410 mutated either to alanine (A) or glutamic acid (E). These mutants shared a diffuse nucleoplasmic pattern with that of fTDP. (B) Mutants of full length TDP with all five serine residues mutated either to alanine (S5A), aspartic acid (S5D) or glutamic acid (S5E) also exhibited a diffuse nucleoplasmic pattern. No significant changes in the number of inclusions were observed. Scale bar in (A) and (B) = 20 µm. (C) Western blot analyses of TDP solubility by sequential extracting Neuro2a cells expressing fTDP, S5A, S5D and S5E with RIPA (R) and urea (U) buffers. As shown, all three proteins were highly soluble evidenced by abundant amounts of proteins in R (soluble) fraction. However, an appreciable increase in the U fraction occurred in S5A sample, suggesting a mild increase in the aggregation propensity. GAPDH was used as loading control. Notably, the S5D and S5E migrated more slowly compared with fTDP and S5A, recapitulating the feature of hyperphosphorylated form of TDP in human ALS/FTLD-U. (D) Filter trap analyses of 20 µg of Neuro2a lysate expressing eGFP, fTDP, S5A or S5E mutant scanned by Typhoon 9410 (GE) to reveal GFP signal. The signal represented aggregated proteins trapped on the cellulose acetate membrane (Toyo Roshi Kaisha, Japan). Notably, the S5E was less trapped than fTDP and S5A. Since fTDP formed aggregates in a small percentage of cells, this finding indicated that hyperphosphorylation-mimetic mutation rendered fTDP less prone for aggregation. (TIF) [file pone.0023075.s003.tif]

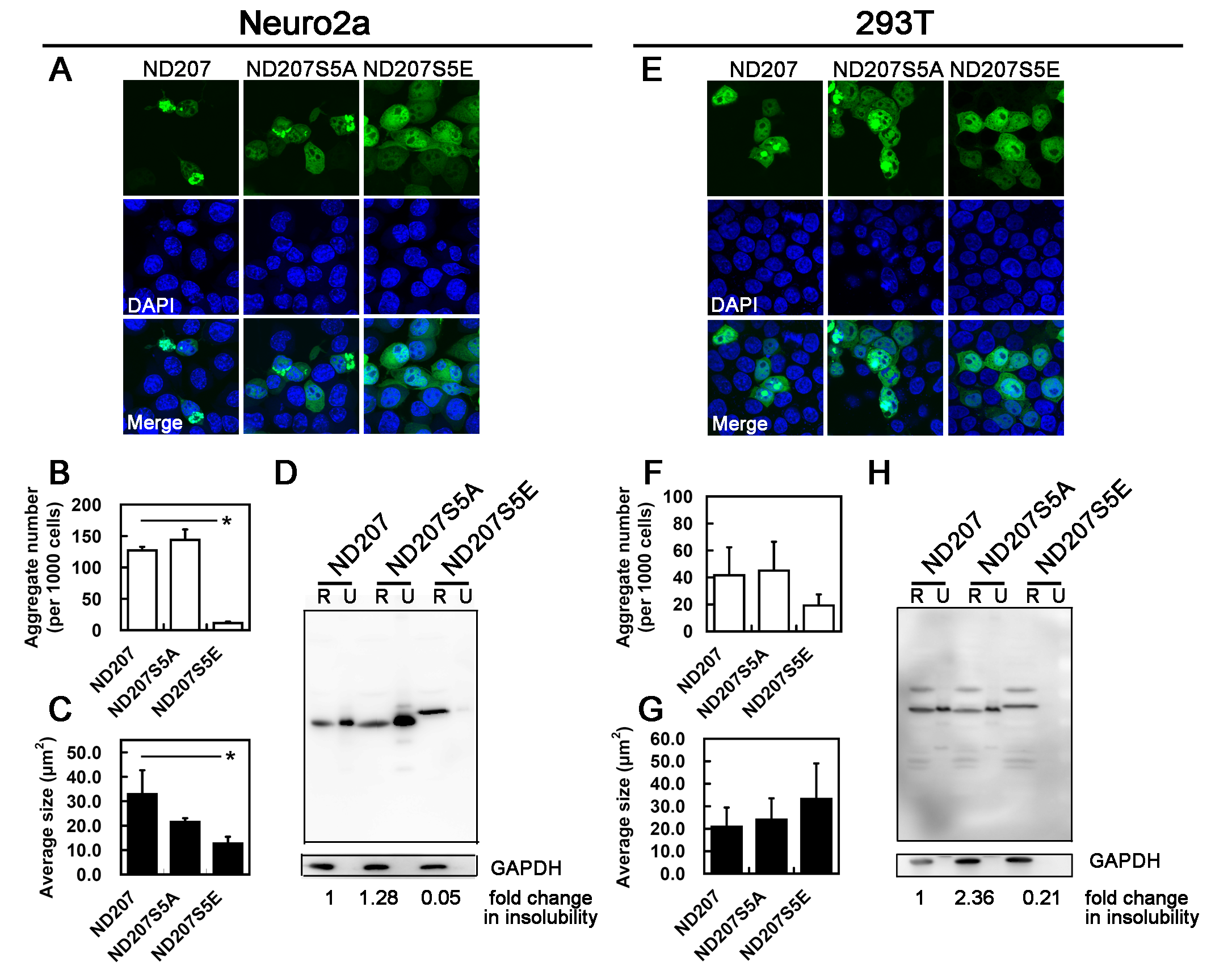

Supplement: Figure S4 — The effect of phosphorylation site mutations on aggregation propensity of ND207. Confocal micrographs of ND207, ND207S5A and ND207S5E in Neuro2a cells (A) or HEK293T cells (E). Scale bar in (A) and (E) = 20 µm. ND207S5E not only formed significantly fewer aggregates in both Neuro2a (B) and HEK293T (F) cells compared with ND207 or ND207S5A, but also significantly impacted the average size of aggregates in Neuro2a cells (C). No significant change in the average size of inclusions was noted in HEK293T cells (G). The solubility of ND207, ND207S5A and ND207S5E in Neuro2a (D) and HEK293T (H) cells were examined by sequential extraction with Western blot. Compared with ND207, ND207S5A was either equally or more insoluble, whereas ND207S5E was highly soluble in RIPA buffer. The fold change of insolubility was calculated with intensity measured by ImageQuant software. (TIF) [file pone.0023075.s004.tif]

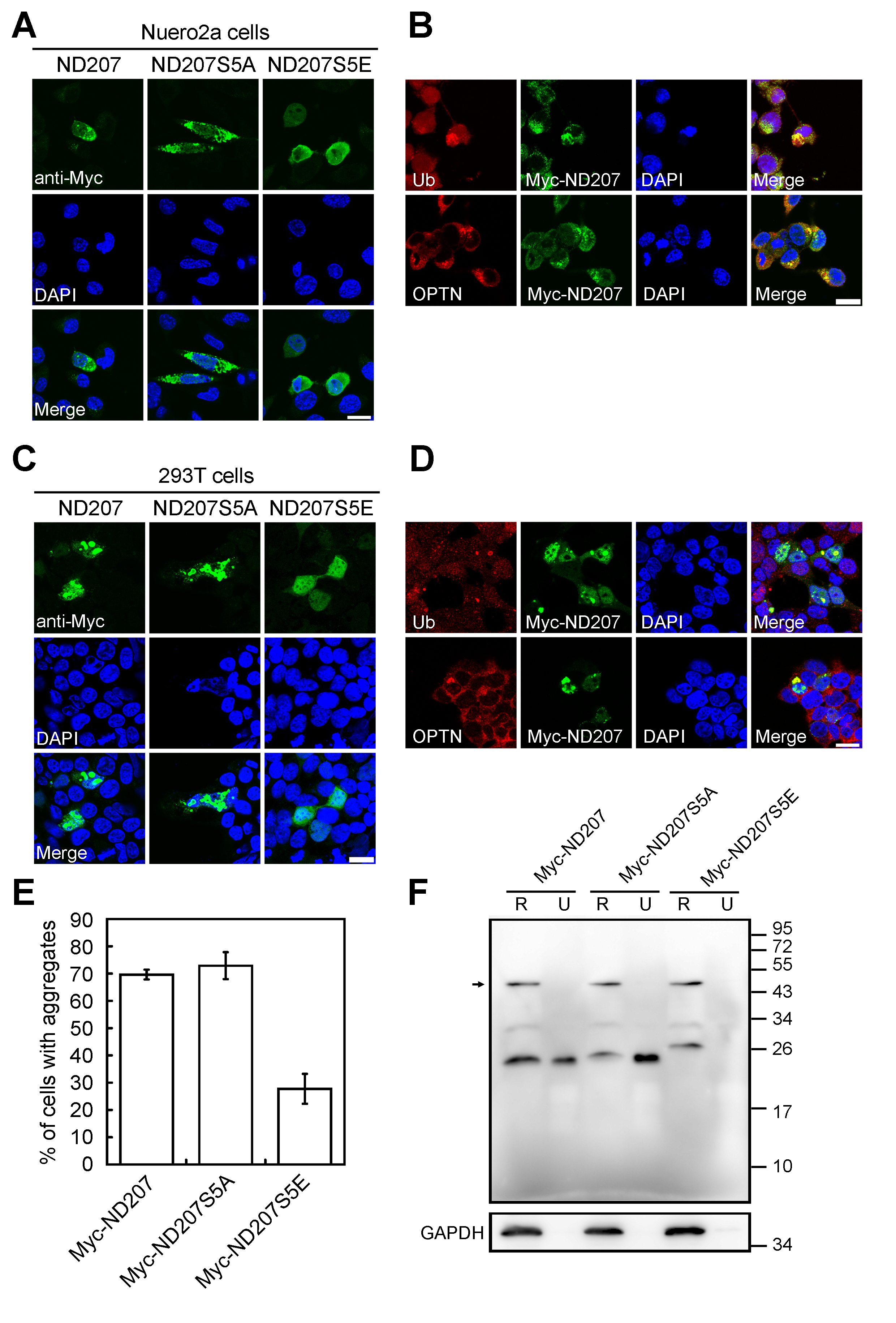

Supplement: Figure S5 — The aggregation propensities of myc tagged ND207, ND207S5A and ND207S5E were similar to those of eGFP-tagged ND207 counterparts. Confocal micrographs of myc tagged ND207, ND207S5A and ND207S5E immunostained by anti-myc antibody in Neuro2a cells (A) or HEK293T cells (C). Scale bar = 20 µm. Similar to eGFP tagged ND207, myc-ND207 also readily formed aggregates which colocalized with both ubiquitin and optineurin signals in Neuro2a cells (B) and HEK293T cells (D). Compared with myc-ND207 or myc-ND207S5A, myc-ND207S5E formed significantly fewer aggregates in HEK293T (E) cells agreed with the quantification done by eGFP-ND207S5E. Moreover, the solubility of ND207, ND207S5A and ND207S5E in HEK293T (H) cells were examined by sequential extraction with Western blot. Compared with ND207, ND207S5A showed a decrease in the solubility (R) fraction, but an increase in the insoluble (U) fraction; whereas, ND207S5E was highly soluble in RIPA buffer. (TIF) [file pone.0023075.s005.tif]

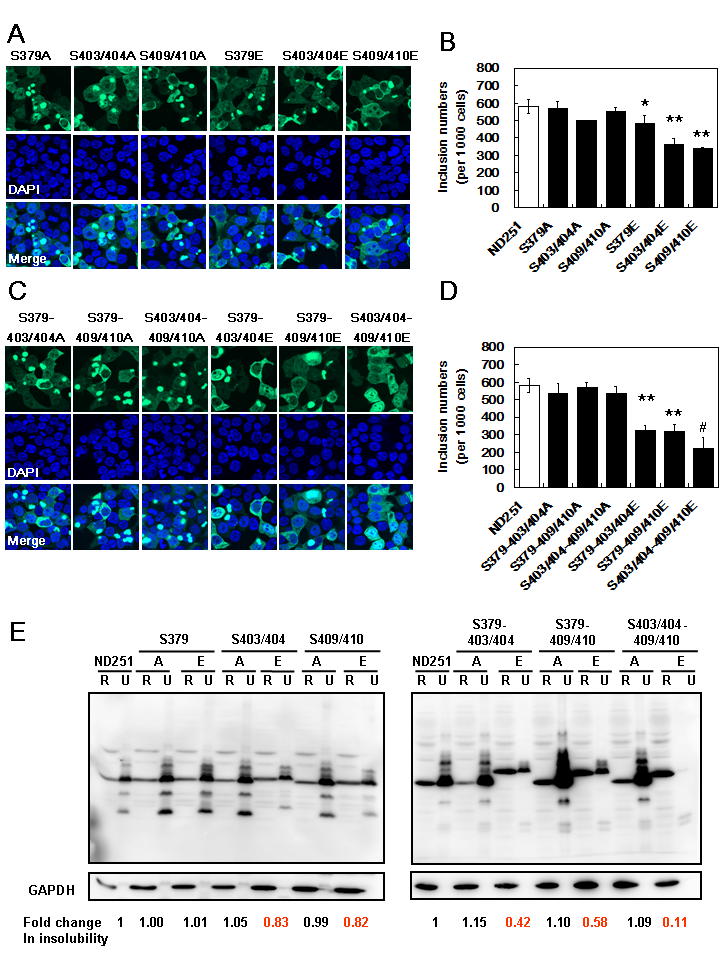

Supplement: Figure S6 — An inverse relationship between the aggregation propensity of ND251 with the number of serine residues mutated to glutamic acids. Confocal micrograph montages of ND251 with single/double mutations (A), and triple/quadruple mutations (C) in HEK293T cells. Scale bar in (A) and (C) = 20 µm. Quantitative analysis (B and D) revealed a trend of gradual decrease in the numbers of inclusions formed by ND251 with the increase in the number of S→E mutations. However, S→A mutants did not exhibit a significant change in the ability to form inclusions. *, P<0.05; **, P<0.005; #, P<0.0005. (E) Consistently, Western blot analysis of the RIPA solubility of the ND251 mutants revealed a consistent increase in solubility with an increase in the number of S→E mutations. The S→A mutations did not significantly alter the solubility of ND251. (TIF) [file pone.0023075.s006.tif]

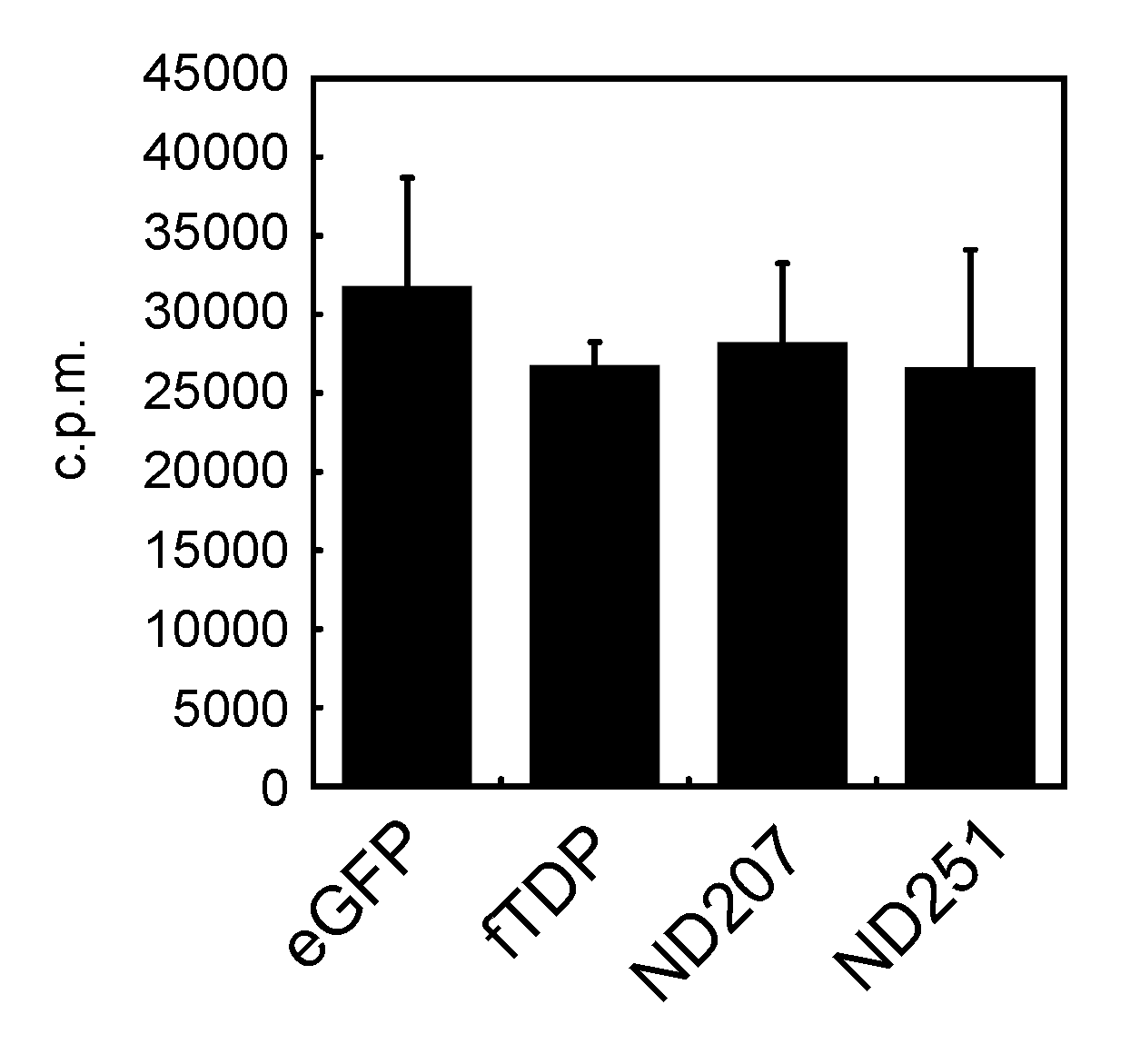

Supplement: Figure S7 — CK2 activities in neuro2a cells were not altered by truncated TDP-43 series overexpression. Endogenous CK2 activity was examined by an in vitro kinase assay employing the measurement of [γ-32P]ATP incorporation into the CK2-specific substrate peptide RRREEETEEE. Neither full length TDP-43 (fTDP) nor truncated TDP-43 series (ND207 or ND251) overexpression changed CK2 activity in neuro2a cells. c.p.m.: counts per minute. (TIF) [file pone.0023075.s007.tif]

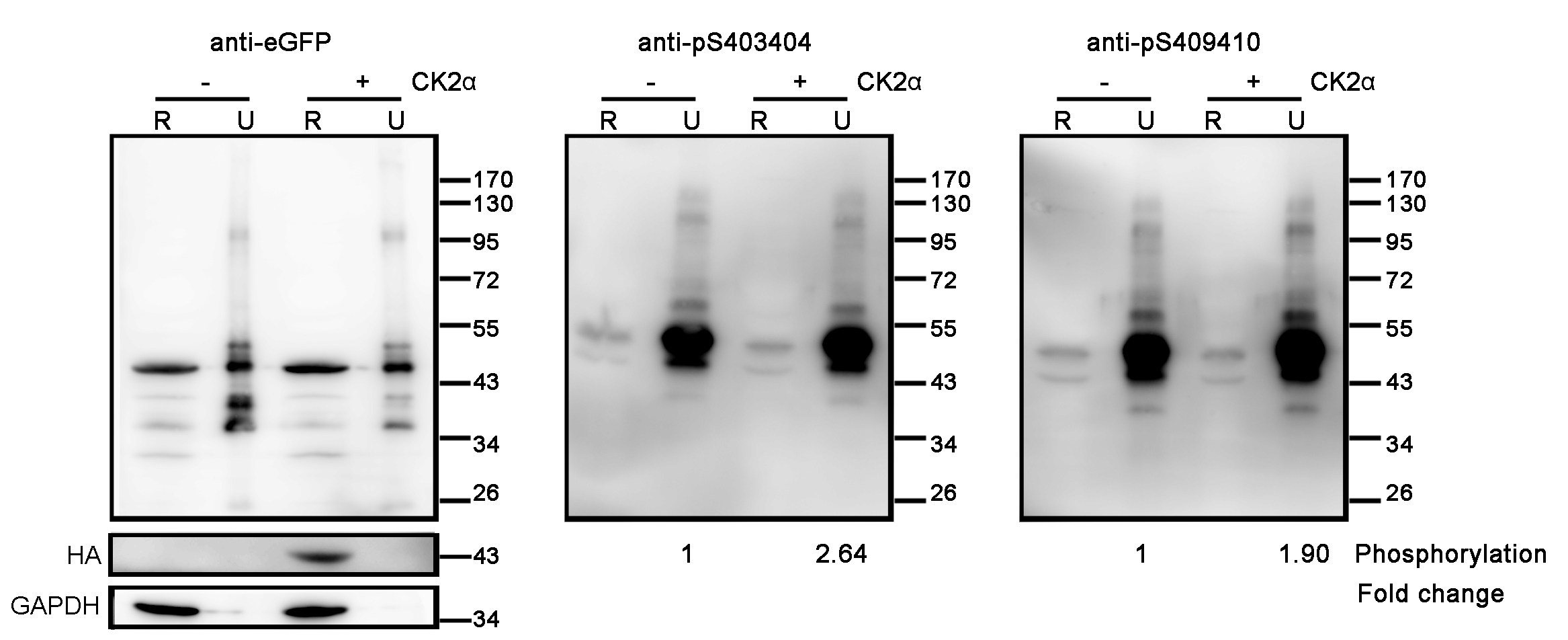

Supplement: Figure S8 — An increase in the phosphorylation status of truncated TDP by CK2α. Expression of CK2α decreased the insoluble ND251 in urea (U) fraction (left panel). However, CK2α increased the phosphorylation status at both serine 403/404 and serine 409/410 residues of the insoluble ND251 in HEK293T cells by ∼2.64 and 1.9 folds, respectively, after normalization. These data supported the notion that hyperphosphorylation decreased the aggregation propensity of ND251. GAPDH was used as loading control and exogenous expression of CK2α was shown with anti-HA (HA) mAb. (TIF) [file pone.0023075.s008.tif]
