# Supplementary material for: Hyperphosphorylation as a Defense Mechanism to Reduce TDP-43 Aggregation
Source: PLoS One. 2011 Aug 5;6(8):e23075. doi: 10.1371/journal.pone.0023075 (PMC3151276; doi:10.1371/journal.pone.0023075)
Supplement: Text S1 — Supplemental methods. (DOC) [file pone.0023075.s009.doc]

**Supplemental methods**

**Generation of Myc tagged ND207**

*pREV-TRE-MycND207* was generated by PCR using *pREV-TRE-emGFPND207* as template with primers: 5’tcggaagaggatctgcgggagttcttctct 3’ and 5’gatgagtttttgttccatggtggcctcgtc 3’.

**Casein kinase 2 activity assay**

Casein Kinase 2 (CK2) activity was measured using the CK2 Assay Kit (Cat #17-132; Millipore, MA, USA) according to manufacturer's instruction. Briefly, 48 hrs after transfections, proteins were extracted by lysis buffer (20mM Tris-Cl pH7.5, 150mM NaCl, 1mM EDTA pH8.0, 1% Triton-X100). 5μg of total protein were incubated at 30°C for 30 minutes in a 25uL reaction mixture containing [γ-32P]ATP and PKA inhibitor cocktail with or without CK2 substrate peptide **(**RRRDDDSDDD). The reaction was stopped by adding 5μL 40% trichloroacetic acid and 25μL were transferred to P81 phosphocellulose squares provided in the kit. After washing 5X with 0.75% phosphoric acid and then 1x with acetone, phosphocellulose squares were dried and transferred to scintillation vials for counting. The kinase activity was determined by subtracting the **background signal of control sample (**without substrate peptide).
